# Multidrug-Resistant *Pseudomonas aeruginosa* Accelerate Intestinal, Extra-Intestinal, and Systemic Inflammatory Responses in Human Microbiota-Associated Mice With Subacute Ileitis

**DOI:** 10.3389/fimmu.2019.00049

**Published:** 2019-01-29

**Authors:** Markus M. Heimesaat, Ulrike Escher, Anne Grunau, Anja A. Kühl, Stefan Bereswill

**Affiliations:** ^1^Gastrointestinal Microbiology Research Group, Institute of Microbiology, Infectious Diseases and Immunology, Berlin Institute of Health, Charité - University Medicine Berlin, Corporate Member of Freie Universität Berlin, Humboldt-Universität zu Berlin, Berlin, Germany; ^2^Department of Medicine I for Gastroenterology, Infectious Diseases and Rheumatology, Research Center ImmunoSciences (RCIS), Berlin Institute of Health, Charité - Universitätsmedizin Berlin, Corporate Member of Freie Universität Berlin, Humboldt-Universität zu Berlin, Berlin, Germany

**Keywords:** multi-drug resistant *Pseudomonas aeruginosa*, subacute ileitis, susceptibility to infection, host-pathogen-interaction, human microbiota associated mice

## Abstract

The globally rising incidences of multidrug-resistant (MDR) *Pseudomonas aeruginosa* (Psae) in humans and live-stock animals has prompted the World Health Organization to rate MDR Psae as serious threat for human health. Only little is known, however, regarding factors facilitating gastrointestinal Psae-acquisition by the vertebrate host and subsequently induced inflammatory sequelae. In the present study, we addressed whether subacute ileitis predisposed mice harboring a human gut microbiota for intestinal MDR Psae carriage and whether inflammatory responses might be induced following peroral challenge with the opportunistic pathogen. To accomplish this, secondary abiotic mice were associated with a human gut microbiota by fecal microbiota transplantation. Ten days later (i.e., on day 0), subacute ileitis was induced in human microbiota associated (hma) mice by peroral low-dose *Toxoplasma gondii* infection. On day 5 post-infection, mice were perorally challenged with 10^9^ colony forming units of a clinical MDR Psae isolate by gavage and the fecal bacterial loads surveyed thereafter. Four days post-peroral challenge, only approximately one third of mice with a human gut microbiota and subacute ileitis harbored the opportunistic pathogen in the intestinal tract. Notably, the gut microbiota composition was virtually unaffected by the Psae-carriage status during subacute ileitis of hma mice. The Psae challenge resulted, however, in more pronounced intestinal epithelial apoptotic cell and T lymphocyte responses upon ileitis induction that were not restricted to the ileum, but also affected the large intestines. Higher Psae-induced abundances of T cells could additionally be observed in extra-intestinal compartments including liver, kidney, lung, and heart of hma mice with subacute ileitis. Furthermore, higher apoptotic cell numbers, but lower anti-inflammatory IL-10 concentrations were assessed in the liver of Psae as compared to mock treated mice with ileitis. Remarkably, Psae-challenge was accompanied by even more pronounced systemic secretion of pro-inflammatory cytokines such as TNF and IL-6 at day 9 post ileitis induction. In conclusion, whereas in one third of hma mice with subacute ileitis Psae could be isolated from the intestines upon peroral challenge, the opportunistic pathogen was responsible for inflammatory sequelae in intestinal, extra-intestinal, and even systemic compartments and thus worsened subacute ileitis outcome irrespective of the Psae-carrier status.

## Introduction

*Pseudomonas aeruginosa* (Psae) constitute Gram-negative, non-fermenting bacteria that grow under strictly aerobic conditions and can be preferably found in moist environments (1). Bacterial motility and adhesive properties are provided by a single flagellum and several cell surface pili. A plethora of additional virulence factors such as alginate secretion, biofilm formation, quorum-sensing, an elaborate secretion system as well as intrinsic expression of antibiotic resistance genes facilitate adaptation to the respective environment as well as immune escape and establishment in the ecosystem of the vertebrate host (1, 2). Psae are considered opportunistic pathogens, but may cause urinary tract infections, ventilator-associated pneumonia, surgical and burn wound as well as bloodstream infections, particularly in hospitalized individuals with immuno-suppressive comorbidities such as neutropenia that are associated with mortality rates of >30% (3). Furthermore, patients suffering from chronic respiratory morbidities including chronic obstructive lung disease, bronchiectasis, or cystic fibrosis are prone to chronic Psae infection, further exacerbating the underlying disease (3). Particularly the emergence of multidrug-resistant (MDR) Psae strains due to bacterial expression of resistance genes such as β-lactamases, 16S rRNA methylases, and carbapenemases in recent years leading to severe infections with high mortality rates due to very limited treatment options has prompted the World Health Organization (WHO) in 2017 to rate MDR Gram-negative bacteria including Psae as serious global threats to human health, further emphasizing the need for novel (antibiotics-independent) treatment strategies (4).

In the healthcare setting, contaminated devices including respiratory equipment, but also water bottles and sinks are typical external sources for Psae acquisition (5, 6). Whereas, Psae is not considered as part of the commensal gut microbiota, a previous report revealed that rectal opportunistic bacterial carriage was a predictor for Psae-associated infections in patients admitted to an intensive care unit (ICU) lateron (7). Thus, the human gastrointestinal tract (GIT) should be taken into consideration as a potential internal Psae reservoir. Particularly in cases, when the integrity of the complex gut microbiota is disturbed following antimicrobial therapy, for instance, the compromised colonization resistance [physiologically preventing the host from establishment of (opportunistic) pathogens (8, 9)] may facilitate stable Psae colonization within the human gastrointestinal ecosystem (10, 11). In an earlier report, Psae could be isolated from fecal samples of healthy volunteers up to 1 week following ingestion with decreasing loads over time, whereas the challenged individuals did not exhibit any clinical complications (12). Valid information regarding the immunopathological potential of intestinal Psae colonization in the vertebrate host is scarce, however.

Recently, our group provided evidence for the first time that intestinal carriage of a clinical MDR Psae isolate resulted in pronounced pro-inflammatory sequelae in intestinal as well as systemic compartments of healthy wildtype (WT) mice (13, 14). Furthermore, pre-existing inflammatory conditions in the gut facilitated stable intestinal Psae colonization in mice (15, 16), which subsequently aggravated chronic colitis (16). In the present study we addressed whether peroral challenge of mice suffering from more acute intestinal inflammatory conditions in a different anatomic compartment within the GIT (namely, subacute ileitis) worsened the outcome of the underlying disease. Since the host specific gut microbiota is known to be essentially involved in the onset, progress and outcome of distinct immunopathological conditions including intestinal inflammation (8, 9, 17, 18), we aimed at mimicking human gut microbiota conditions in the applied experimental model. We therefore generated mice that were harboring a human gut microbiota following human fecal microbiota transplantation (FMT) and induced subacute ileitis by peroral low-dose *Toxoplasma gondii* infection as described by us recently (19, 20). We here show that approximately one third of human microbiota associated (hma) mice with subacute ileitis harbored Psae in their intestines upon oral challenge. The opportunistic pathogen was, however, responsible for pro-inflammatory immune responses in intestinal, extra-intestinal and even systemic compartments and, thus, worsened subacute ileitis outcome irrespective of the Psae-carrier status.

## Materials and Methods

### Ethics Statement

Mouse experiments were performed in accordance of the European Guidelines for animal welfare (2010/63/EU) and had been approved by the commission for animal experiments headed by the “Landesamt für Gesundheit und Soziales” (LaGeSo, Berlin; registration numbers G0097/12 and G0039/15) before. Clinical conditions including body weight loss were assessed daily to assure animal welfare. Mice displaying over 20% weight loss were subjected to cervical dislocation in accordance with the guidelines of the local legal authorities.

### Generation of Mice With a Human Gut Microbiota by Fecal Microbiota Transplantation

Female C57BL/6j mice were maintained under specific pathogen-free (SPF) conditions in the Forschungseinrichtungen für Experimentelle Medizin (FEM, Charité - University Medicine, Berlin, Germany). Microbiota depleted (i.e., secondary abiotic) mice were obtained following broad-spectrum antibiotic treatment as described earlier (8, 17). In brief, 8-week-old mice were treated with a quintuple antibiotic cocktail containing ampicillin plus sulbactam (1 g/L; Ratiopharm, Germany), vancomycin (500 mg/L; Cell Pharm, Germany), ciprofloxacin (200 mg/L; Bayer Vital, Germany), imipenem (250 mg/L; MSD, Germany), and metronidazole (1 g/L; Fresenius, Germany) via the drinking water for 8 weeks (*ad libitum*). Both, culture and 16S rRNA based molecular methods revealed virtual absence of bacteria in fecal samples as described previously (8, 18). Three days before human FMT the antibiotic cocktail was replaced by sterile tap water (*ad libitum*) to assure antibiotic washout (Supplemental Figure S1). Fresh fecal samples free of enteropathogenic bacteria, viruses, and parasites were collected from five individual healthy human volunteers, dissolved in sterile phosphate buffered saline (PBS; Gibco, Life Technologies, United Kingdom) and stored at −80°C as stated elsewhere (8). Individual fecal aliquots were thawed immediately before FMT and pooled. In turn, secondary abiotic mice were perorally challenged with the human fecal donor suspension on three consecutive days by gavage (8). Notably, the inter-experimental variations in bacterial loads of the donor suspensions were < 0.5 logarithmic orders of magnitude (Supplementary Figure S2). In order to guarantee proper establishment of the complex human gut microbiota within the murine host, mice were kept for 10 days before subacute ileitis induction. Immediately before peroral *T. gondii* infection (day 0) individual fecal samples were collected for quantitative molecular analyses of main intestinal bacterial communities as described elsewhere (8, 17, 21).

### Molecular Analysis of the Human Fecal Donor Suspensions and the Intestinal Microbiota

Fresh ileal and colonic luminal samples were immediately snap-frozen in liquid nitrogen and stored at −80°C until further processing. DNA was extracted from fecal samples as reported earlier (17). In brief, for quantification of DNA the Quant-iT PicoGreen reagent (Invitrogen, UK) was applied and DNA in samples adjusted to 1 ng per μL. Then, total eubacterial loads as well as the main bacterial groups within the human gut microbiota such as enterobacteria, enterococci, lactobacilli, bifidobacteria, *Bacteroides/Prevotella* species, *Clostridium coccoides* group, and *Clostridium leptum* group were surveyed by quantitative real-time polymerase chain reaction using genera-, group- or species-specific 16S rRNA gene primers (Tib MolBiol, Germany) as stated elsewhere (21, 22) and numbers of 16S rRNA gene copies per ng DNA of each sample assessed.

### Induction of Subacute Ileitis

On day 0, mice were infected perorally with one cyst of *T. gondii* ME49 strain (in a volume of 0.3 mL brain suspension) by gavage in order to induce subacute ileitis (Supplemental Figure S1), as reported recently (19).

### MDR *P. aeruginosa* Infection and Quantitative Assessment of Fecal Bacterial Loads

The MDR Psae isolate was initially isolated from respiratory material of a patient with nosocomial pneumonia and kindly provided by Prof. Dr. Bastian Opitz (Charité–University Medicine, Berlin, Germany). Notably, the bacterial strain displayed antimicrobial sensitivity to fosfomycin and colistin only (15). Prior infection, the Psae strain was grown on cetrimide agar (Oxoid) for 48 h in an aerobic atmosphere at 37°C.

On day 5 post ileitis induction, mice were perorally challenged with 10^9^ colony forming units (CFU) of the MDR Psae strain by gavage in a total volume of 0.3 mL PBS as reported earlier (Supplemental Figure S1) (15).

For quantitative assessment of Psae colonization densities in the intestinal tract post-challenge, fecal and intestinal luminal samples were homogenized in sterile PBS. Serial dilutions were then streaked onto Columbia agar supplemented with 5% sheep blood (Oxoid, Germany) and onto cetrimide agar and incubated in an aerobic atmosphere at 37°C for 48 h as described previously (15). Difference of the sample weights before and after asservation revealed respective fecal weights. The detection limit of cultivable bacteria was ~100 CFU per g.

### Cultural Survey of Bacterial Translocation

In order to survey viable bacteria translocating from the intestinal tract to extra-intestinal and systemic compartments, *ex vivo* biopsies derived from mesenteric lymph nodes (MLN), spleen, liver, kidney and lung were homogenized in sterile PBS and analyzed in serial dilutions on respective solid media as described earlier (17, 23, 24). Cardiac blood was incubated in thioglycolate enrichment broths (BD Bioscience, Germany) for 1 week at 37°C and streaked onto solid media thereafter (15). Bacteria were grown at 37°C for at least two and t3 days under aerobic, microaerobic and anaerobic conditions.

### Clinical Conditions

Macroscopic and / or microscopic occurrence of blood in murine fecal pellets was surveyed in individual mice on a daily basis by the Guajac method (Haemoccult; Beckman Coulter/ PCD, Germany) as stated elsewhere (25).

### Sampling Procedures

96 h following Psae challenge (i.e., day 9 post ileitis induction), mice were sacrificed by isoflurane treatment [Abbott, Germany (Figure S1)]. Cardiac blood (for serum) and tissue samples from spleen, liver, lung, heart, MLN, ileum, and colon were removed under sterile conditions. Respective *ex vivo* biopsies were collected from each mouse in parallel for microbiological, immunological and immunohistochemical analyses.

### Histopathology

*Ex vivo* biopsies taken from the terminal ileum were fixed in 5% formalin and embedded in paraffin. Sections (5 μm) were stained with hematoxylin and eosin (H&E) and subjected to a standardized histopathological scoring system ranging from 0 to 6 as described in more detail earlier (17).

### Immunohistochemistry

Five μm thin paraffin sections of ileal and colonic *ex vivo* biopsies were used for *in situ* immunohistochemical analysis as reported previously (21, 26–28). In brief, to assess apoptotic cells, proliferating cells, T lymphocytes and regulatory T cells (Treg), primary antibodies against cleaved caspase-3 (Asp175, #9661, Cell Signaling, Leiden, Netherlands; 1:200), Ki67 (clone 16A8, #652401, BioLegend/Biozol, Eching, Germany; 1:200), CD3 (#IR50361-2, Dako, Santa Clara, CA, USA; 1:5) and FOXP3 (clone FJK-165, #14-5773, eBioscience, Frankfurt, Germany; 1:100), respectively, were used. An independent blinded investigator determined the average numbers of positively stained cells in at least six high power fields (HPF, 0.287 mm^2^; 400 x magnification).

### Cytokine Detection

*Ex vivo* biopsies (~1 cm^2^) derived from colon and ileum (both cut longitudinally and washed in PBS), as well as from liver (~1 cm^2^), kidney (cut longitudinally, one half), lung (one organ), spleen (one half), and MLN (3 single lymph nodes) were transferred to 24-flat-bottom well culture plates (Falcon, Germany) containing 500 μL serum-free RPMI 1640 medium (Gibco, life technologies) supplemented with penicillin (100 U/mL, Biochrom, Germany) and streptomycin (100 μg/mL; Biochrom). After 18 h at 37°C, IFN-γ, TNF, MCP-1, IL-6, and IL-10 concentrations were measured in culture supernatants and serum by the Mouse Inflammation Cytometric Bead Assay (CBA; BD Bioscience) applying a BD FACSCanto II flow cytometer (BD Bioscience). Nitric oxide (NO) concentrations were determined by the Griess reaction as described earlier (17).

### Statistical Analysis

Medians and levels of significance were determined by the one-way ANOVA test followed by Tukey post-correction test for multiple comparisons. Two-sided probability (*p*) values ≤ 0.05 were considered significant. Experiments were reproduced three times.

## Results

### Colonization Properties of MDR *P. aeruginosa* in the Intestinal Tract of Mice With a Human Gut Microbiota Suffering From Subacute Ileitis

**Figure 1:**
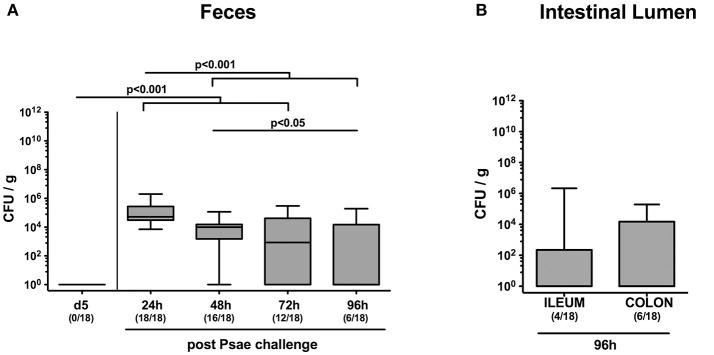
Intestinal MDR *Pseudomoas aeruginosa* loads following peroral challenge of mice with a human gut microbiota suffering from subacute ileitis. Secondary abiotic mice were generated by broad-spectrum antibiotic treatment and associated with a complex human gut microbiota by fecal microbiota transplantation. Ten days thereafter, subacute ileitis was induced by peroral infection of human microbiota associated mice with one cyst of *T. gondii* ME49 strain (day 0). On day 5 post ileitis induction (p.i.), mice were perorally challenged with MDR *P. aeruginosa* (Psae) and **(A)** the fecal Psae loads were followed up for further 96 h by culture. **(B)** In addition, Psae counts were assessed in the small as well as large intestinal lumen 96 h post Psae challenge. Box plots represent the 75th and 25th percentiles of the median (black bar inside the boxes). Total range and significance levels determined by one-way ANOVA test followed by Tukey post-correction test for multiple comparisons are shown. Numbers of mice harboring Psae in the respective intestinal compartment out of the total numbers of analyzed animals are given in parentheses. Data were pooled from four independent experiments.

In the present study we raised the question whether subacute ileitis predisposed mice with a human gut microbiota for intestinal MDR Psae carriage and whether intestinal, extra-intestinal or even systemic inflammatory sequelae might be induced following peroral Psae challenge. To address this, secondary abiotic mice were associated with a complex human gut microbiota by FMT on three consecutive days. Ten days later (i.e., on day 0), subacute ileitis was induced by peroral low-dose *T. gondii* infection of hma mice. On day 5 post ileitis induction, mice were perorally challenged with 10^9^ CFU of a clinical MDR *P. aeruginosa* isolate by gavage and the fecal opportunistic pathogenic loads surveyed thereafter (Supplemental Figure S1). As early as 24 h following peroral challenge, fecal Psae could be cultured from all mice with median loads of ~10^5^ CFU per g (Figure 1A). At later time points (48, 72, and 96 h, respectively), however, both fecal Psae positivity rates (88.9, 66.7, and 33.3%, respectively) as well as median Psae loads (*p* < 0.001; Figure 1A) were lower, whereas at the end of the experiment, Psae could be isolated from the ileal and colonic lumen in 22.2% and 33.3% of cases, respectively, (Figure 1B). Hence, only one third of mice with a human gut microbiota and subacute ileitis harbored MDR Psae in their intestinal tract 4 days post peroral challenge.

### Gut Microbiota Changes in Mice With a Human Gut Microbiota and Subacute Ileitis Following MDR *P. aeruginosa* Challenge

**Figure 2:**
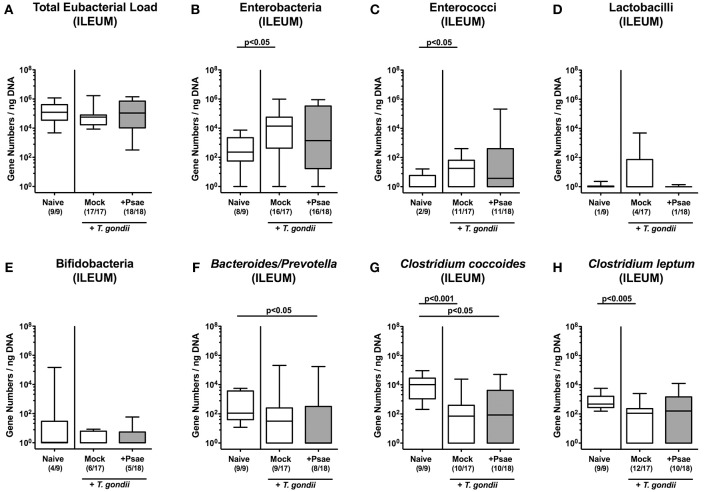
Ileal microbiota changes following MDR *P. aeruginosa* challenge of mice with a human gut microbiota suffering from subacute ileitis. Subacute ileitis was induced in mice harboring a human gut microbiota by peroral *T. gondii* infection on day (d) 0. At day 5 post ileitis induction mice were either perorally challenged with MDR *P. aeruginosa* (+Psae, dissolved in PBS) or with PBS alone (Mock). Uninfected mice with a human gut microbiota but without ileitis served as control animals (Naive). **(A–H)** The microbiota composition of the ileal lumen was determined 96 h later by quantitative Real-Time PCR amplifying bacterial 16S rRNA variable regions of the main intestinal bacterial groups (expressed as 16S rRNA gene numbers per ng DNA) including the total eubacterial load. Box plots represent the 75th and 25th percentiles of the median (black bar inside the boxes). Total range and significance levels determined by one-way ANOVA test followed by Tukey post-correction test for multiple comparisons are shown. Numbers of mice harboring respective bacterial groups in the ileal lumen out of the total numbers of analyzed animals are given in parentheses. Data were pooled from four independent experiments.

We next addressed whether MDR Psae challenge following subacute ileitis induction resulted in intestinal microbiota changes in mice with a human gut microbiota (Figures 2A–H). Quantitative culture-independent analysis revealed that until day 9 following ileitis induction mock treated hma mice displayed increased ileal loads of enterobacteria and enterococci (*p* < 0.05; Figures 2B,C), whereas both ileal *Clostridium coccoides* and *Clostridium leptum* group gene numbers were lower as compared to naive hma mice without ileitis (*p* < 0.001 and *p* < 0.005, respectively; Figures 2G,H). At day 9 post ileitis induction, lower *Bacteroides / Prevotella* species and *Clostridium coccoides* group loads could be detected in the ileal lumen of Psae challenged as compared to naive mice (*p* < 0.05; Figures 2F,G).

**Figure 3:**
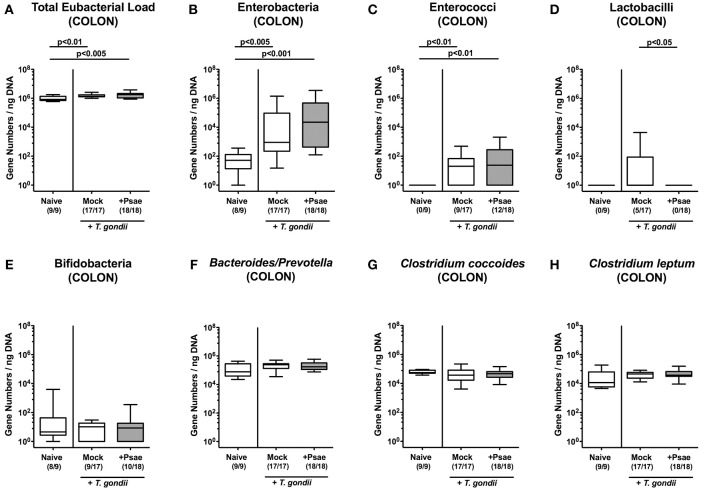
Colonic microbiota changes following MDR *P. aeruginosa* challenge of mice with a human gut microbiota suffering from subacute ileitis. Subacute ileitis was induced in mice harboring a human gut microbiota by peroral *T. gondii* infection on day (d) 0. At day 5 post ileitis induction mice were either perorally challenged with MDR *P. aeruginosa* (+Psae, dissolved in PBS) or with PBS alone (Mock). Uninfected mice with a human gut microbiota but without ileitis served as control animals (Naive). **(A–H)** The microbiota composition of the colonic lumen was determined 96 h later by quantitative Real-Time PCR amplifying bacterial 16S rRNA variable regions of the main intestinal bacterial groups (expressed as 16S rRNA gene numbers per ng DNA) including the total eubacterial load. Box plots represent the 75th and 25th percentiles of the median (black bar inside the boxes). Total range and significance levels determined by one-way ANOVA test followed by Tukey post-correction test for multiple comparisons are shown. Numbers of mice harboring respective bacterial groups in the colonic lumen out of the total numbers of analyzed animals are given in parentheses. Data were pooled from four independent experiments.

We additionally assessed the microbiota composition in the large intestinal tract (Figures 3A–H). Within 9 days following ileitis induction, total eubacterial loads increased in the colonic lumen of both mock and Psae challenged mice (*p* < 0.01 and *p* < 0.005, respectively; Figure 3A). This also held true for large intestinal enterobacteria (*p* < 0.005 and *p* < 0.001, respectively; Figure 3B) and enterococci (*p* < 0.01; Figure 3C), whereas Psae challenged animals displayed lower lactobacilli loads in their colon as compared to mock treated controls at day 9 p.i. (*p* < 0.05; Figure 3D). Hence, the intestinal loads of assessed bacterial taxa, groups and species did not differ between Psae challenged and unchallenged hma mice during subacute ileitis.

### Macroscopic and Microscopic Inflammatory Sequelae of MDR *P. aeruginosa* Challenge in Mice With a Human Gut Microbiota Suffering From Subacute Ileitis

**Figure 4:**
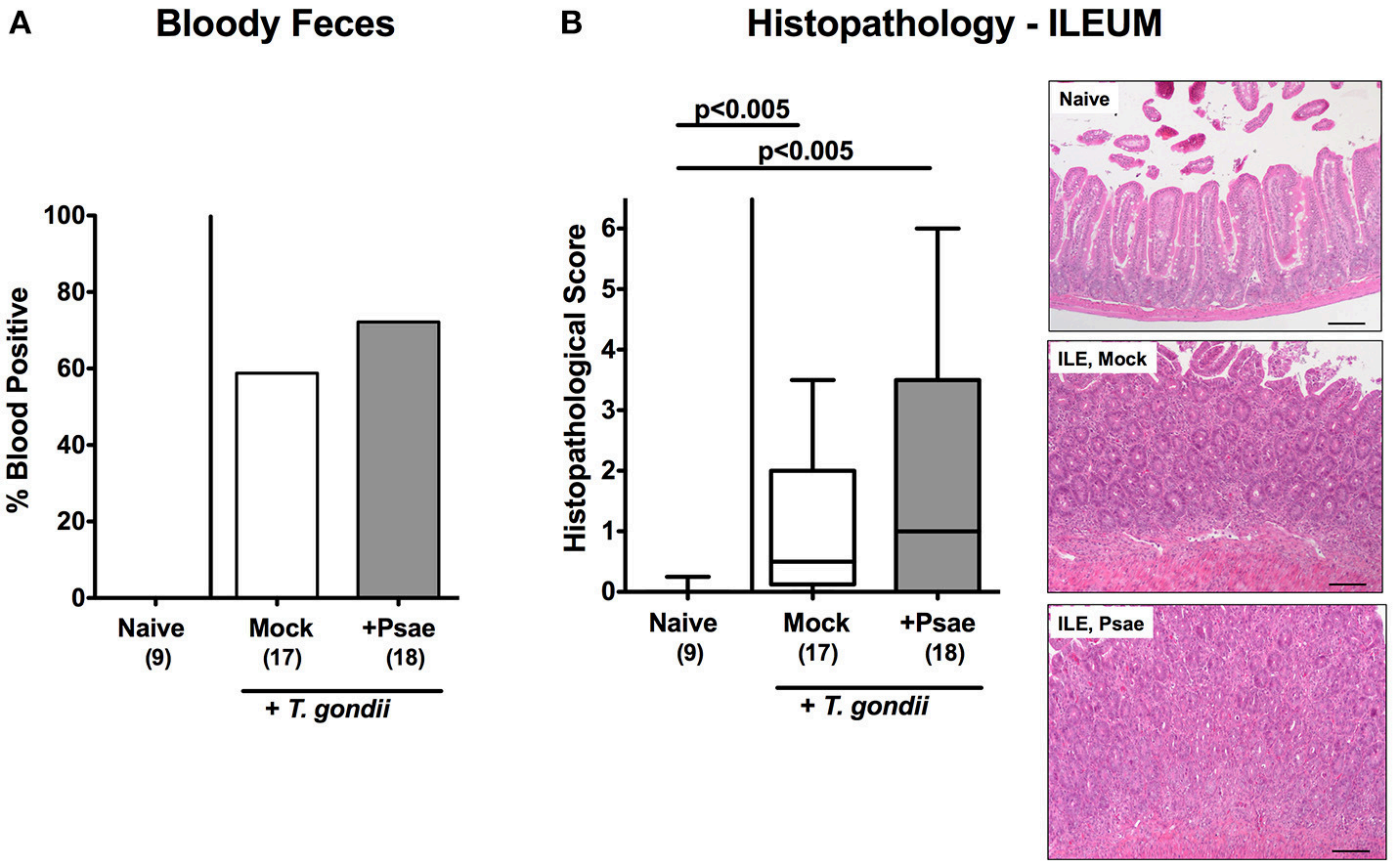
Clinical and histopathological sequelae following peroral MDR *P. aeruginosa* challenge of mice with a human gut microbiota suffering from subacute ileitis. At day 5 following subacute ileitis induction, mice with a human gut microbiota were either perorally challenged with MDR *P. aeruginosa* (+Psae, dissolved in PBS) or with PBS alone (Mock). Uninfected mice with a human gut microbiota but without ileitis served as control animals (Naive). Clinical and microscopic intestinal changes were assessed 96 h following Psae challenge. **(A)** Abundance of blood was determined in fecal samples by the Guajac (Haemoccult) method. Cumulative blood positivity rates were calculated from four independent experiments. **(B)** Histopathological changes were determined in H&E stained ileal paraffin sections applying a standardized scoring system (see methods) and representative photomicrographs depicted (right panel; 100x magnification, scale bar 100 μm). Box plots represent the 75th and 25th percentiles of the median (black bar inside the boxes). Total range and significance levels determined by one-way ANOVA test followed by Tukey post-correction test for multiple comparisons are shown. The total numbers of analyzed animals are given in parentheses. Data were pooled from four independent experiments.

We next assessed whether peroral Psae challenge worsened subacute ileitis of hma mice. Mice of either treatment group displayed mean body weight loss of ~5% during the observation period (not shown). At day 9 p.i., however, 58.8% of mock treated mice exhibited blood in their feces, whereas this was the case in 72.2% of Psae challenged and none of naive mice (Figure 4A). Following ileitis induction, mice of either cohort displayed comparable histopathological changes within the small intestinal mucosa and lamina propria (*p* < 0.005 vs. naive controls; Figure 4B). Of note, the range of histopathological scores was higher following Psae as compared to mock challenged mice at day 9 p.i., with single mice displaying even ileal necrosis in the former cohort, which was not the case in the latter group (Figure 4B).

### Intestinal Apoptotic, Proliferating, and Immune Cell Responses Upon MDR *P. aeruginosa* Challenge of Mice With a Human Gut Microbiota Suffering From Subacute Ileitis

**Figure 5:**
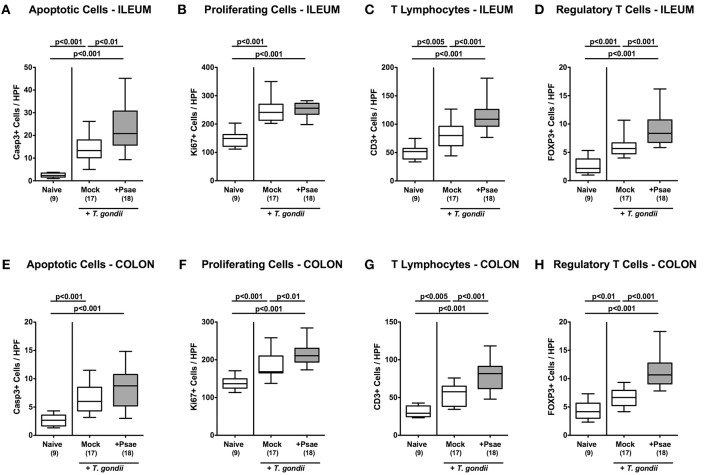
Apoptotic and proliferating epithelial cell as well as immune cell responses in the ileum and colon following peroral MDR *P. aeruginosa* challenge of mice with a human gut microbiota suffering from subacute ileitis. At day 5 following subacute ileitis induction, mice with a human gut microbiota were either perorally challenged with MDR *P. aeruginosa* (+Psae, dissolved in PBS) or with PBS alone (Mock). Uninfected mice with a human gut microbiota but without ileitis served as control animals (Naive). 96 h following Psae challenge, the average numbers of apoptotic (Casp3+; **A**,**E**) and proliferating (Ki67+; **B**,**F**) epithelial cells as well as of T lymphocytes (CD3+; **C,G**), and regulatory T cells (FOXP3+; **D,H**) in at least six high power fields (HPF) were quantitatively assessed in ileal **(A–D)** and colonic **(E–H)** paraffin sections applying *in situ* immunhistochemistry. Box plots represent the 75th and 25th percentiles of the median (black bar inside the boxes). The total range and significance levels determined by one-way ANOVA test followed by Tukey post-correction test for multiple comparisons are shown. The total numbers of analyzed animals are given in parentheses. Data were pooled from four independent experiments.

We further expanded our histopathological analyses and quantitatively assessed apoptotic cells in the small intestinal epithelia applying *in situ* imunohistochemistry. Ileitis induction was accompanied by increased numbers of caspase3+ ileal epithelial cells (*p* < 0.001; Figure 5A; Supplemental Figure S3A). Of note, at day 9 p.i., Psae challenged mice were suffering from even more distinct ileal epithelial apoptosis as compared to mock treated mice (*p* < 0.01; Figure 5A; Supplemental Figure S3A).

We further quantitated Ki67+ cells in ileal epithelia indicative for proliferative/regenerative cell responses counteracting potential Psae-induced cell damage. Until day 9 p.i., Ki67+ cell numbers had markedly increased in the ileal epithelia as compared to naive controls (*p* < 0.001; Figure 5B; Supplemental Figure S3B), but irrespective whether mice were Psae or mock challenged (n.s.; Figure 5B; Supplemental Figure S3B).

Given that *T. gondii* induced ileitis comprises a T cell-driven immunopathological scenario (29), we next assessed T lymphocyte counts in ileal paraffin sections by *in situ* imunohistochemistry. At day 9 p.i., *T. gondii* infected mice displayed increased numbers of CD3+ cells in their ileal mucosa and lamina propria, but exhibited even higher T cell counts upon Psae as compared to mock challenge (*p* < 0.001; Figure 5C; Supplemental Figure S3C). Interestingly, increases in small intestinal T cell numbers following ileitis induction were paralleled by elevated numbers of FOXP3+ cells in the ileal mucosa and lamina propria of both, Psae and mock challenged mice at day 9 p.i. (*p* < 0.001; Figure 5D; Supplemental Figure S3D) with higher counts in the former as compared to the latter (*p* < 0.001; Figure 5D; Supplemental Figure S3D).

Even though peroral *T. gondii* infection of susceptible mice is well-known to affect primarily the terminal ileum (29), we additionally included the large intestines into our immunohistopathological survey. Interestingly, ileitis induction was also accompanied by increases in apoptotic cell numbers in colonic epithelia (*p* < 0.001; Figure 5E; Supplemental Figure S3E) with a trend toward even higher counts in Psae as compared to mock treated mice at day 9 p.i. (n.s. due to high standard deviations). Like in the ileum, *T. gondii* infection resulted in increased Ki67+ colonic epithelial numbers (*p* < 0.001; Figure 5F; Supplemental Figure S3F), that were even more pronounced in Psae as compared to mock challenged mice (*p* < 0.01; Figure 5F; Supplemental Figure S3F). Furthermore, *T. gondii* induced elevated numbers of both CD3+ and FOXP3+ cells could be determined in the mucosa and lamina propria of the large intestines at day 9 p.i. (*p* < 0.001; Figures 5G,H; Supplemental Figures S3G,H) with even higher counts in the Psae vs. mock cohort (*p* < 0.001; Figures 5G,H; Supplemental Figures S3G,H). Hence, Psae challenge resulted in more pronounced intestinal epithelial apoptotic and T cell responses upon ileitis induction that were not restricted to the ileum, but also involved the large intestines.

### Intestinal Pro-Inflammatory Mediator Responses Upon MDR *P. aeruginosa* Challenge of Mice With a Human Gut Microbiota Suffering From Subacute Ileitis

We next addressed whether changes in apoptotic and immune cell responses upon Psae challenge of hma mice with subacute ileitis was accompanied by pro-inflammatory mediator responses in the intestinal tract. Irrespective whether Psae or mock challenged, hma mice displayed increased IFN-γ concentrations in *ex vivo* biopsies derived from the ileum, colon or MLN at day 9 p.i. (*p* < 0.001 vs. naive controls; Supplemental Figures S4A–C). In addition, TNF and NO were multi-fold elevated in MLN following ileitis induction in either cohort (*p* < 0.005–0.001 vs. naive; Supplemental Figures S4D,E). Hence, Psae challenge did not result in further augmented pro-inflammatory mediator responses in the intestinal tract following subacute ileitis induction of hma mice.

### Extra-Intestinal Apoptotic, Proliferating, and Immune Cell Responses Upon MDR *P. aeruginosa* Challenge of Mice With a Human Gut Microbiota Suffering From Subacute Ileitis

**Figure 6:**
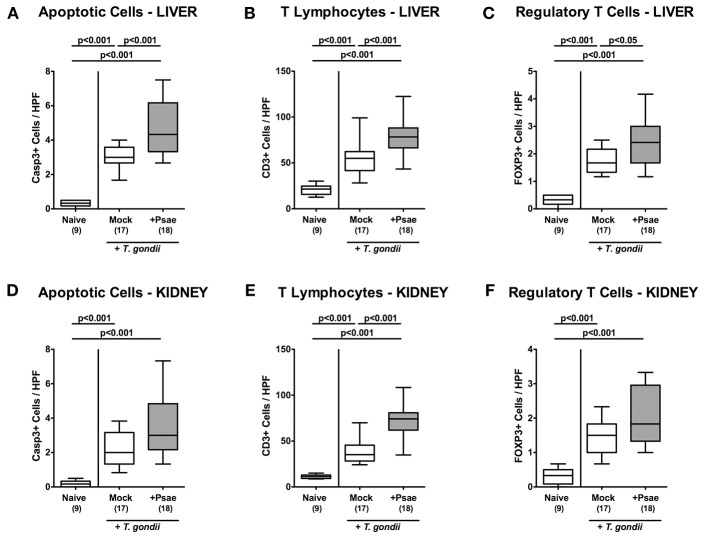
Apoptotic epithelial cell as well as immune cell responses in the liver and kidney following peroral MDR *P. aeruginosa* challenge of mice with a human gut microbiota suffering from subacute ileitis. At day 5 following subacute ileitis induction, mice with a human gut microbiota were either perorally challenged with MDR *P. aeruginosa* (+Psae, dissolved in PBS) or with PBS alone (Mock). Uninfected mice with a human gut microbiota but without ileitis served as control animals (Naive). 96 h following Psae challenge, the average numbers of apoptotic (Casp3+; **A,D**) epithelial cells as well as of T lymphocytes (CD3+; **B,E**) and regulatory T cells (FOXP3+; **C,F**) in at least six high power fields (HPF) were quantitatively assessed in hepatic **(A–C)** and renal **(D–F)** paraffin sections applying *in situ* immunhistochemistry. Box plots represent the 75th and 25th percentiles of the median (black bar inside the boxes). The total range and significance levels determined by one-way ANOVA test followed by Tukey post-correction test for multiple comparisons are shown. The total numbers of analyzed animals are given in parentheses. Data were pooled from four independent experiments.

We next addressed whether peroral MDR Psae challenge of hma mice with subacute ileitis might lead to more pronounced inflammatory responses in extra-intestinal compartments including liver, kidney, lung, and heart. Nine days following ileitis induction, hma mice exhibited increased numbers of apoptotic hepatic cells (*p* < 0.001 vs. naive; Figure 6A; Supplemental Figure S5A) that were even higher upon Psae as compared to mock challenge (*p* < 0.001; Figure 6A; Supplemental Figure S5A). Increases in apoptotic cells were paralleled by elevated T lymphocyte and Treg counts in the livers of both, Psae and mock challenged mice at day 9 p.i. (*p* < 0.001; Figures 6B,C; Supplemental Figures S5B,C with higher numbers in the former as compared to the latter (*p* < 0.001 and *p* < 0.05, respectively; Figures 6B,C; Supplemental Figures S5B,C.

In the kidney, apoptotic cells, CD3+ and FOXP3+ cells increased upon ileitis induction of hma mice until day 9 p.i. (Figures 6D–F, Supplemental Figures S5D–F). Additional Psae challenge resulted in more pronounced increases in renal T lymphocytes as compared to the mock cohort (Figure 6E; Supplemental Figure S5E), whereas a trend toward higher Casp3+ and FOXP3+ cell counts could be observed in the kidneys following Psae challenge at day 9 p.i. (n.s.; Figures 6D,F, Supplemental Figures S5D,F).

**Figure 7:**
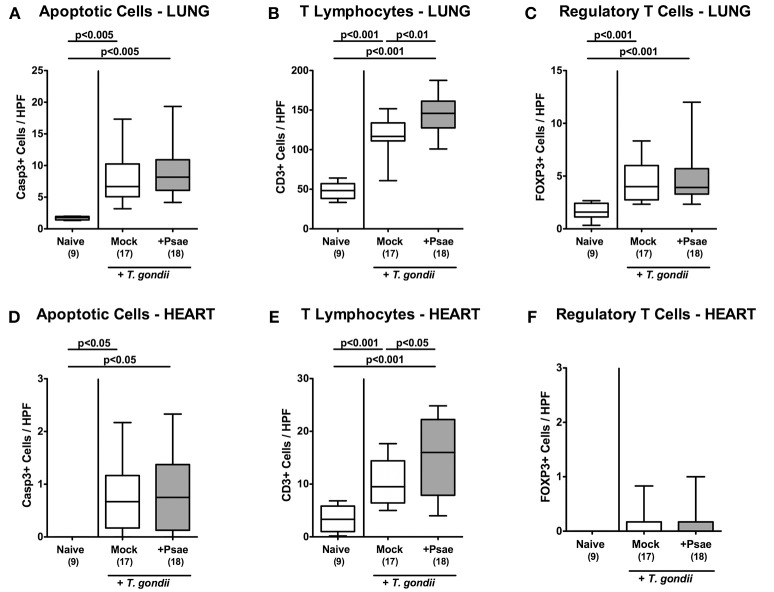
Apoptotic epithelial cell as well as immune cell responses in the lung and heart following peroral MDR *P. aeruginosa* challenge of mice with a human gut microbiota suffering from subacute ileitis. At day 5 following subacute ileitis induction, mice with a human gut microbiota were either perorally challenged with MDR *P. aeruginosa* (+Psae, dissolved in PBS) or with PBS alone (Mock). Uninfected mice with a human gut microbiota but without ileitis served as control animals (Naive). 96 h following Psae challenge, the average numbers of apoptotic (Casp3+; **A,D**) epithelial cells as well as of T lymphocytes (CD3+; **B,E**) and regulatory T cells (FOXP3+; **C,F**) in at least six high power fields (HPF) were quantitatively assessed in pulmonal **(A–C)** and cardiac **(D–F)** paraffin sections applying *in situ* immunhistochemistry. Box plots represent the 75th and 25th percentiles of the median (black bar inside the boxes). The total range and significance levels determined by one-way ANOVA test followed by Tukey post-correction test for multiple comparisons are shown. The total numbers of analyzed animals are given in parentheses. Data were pooled from four independent experiments.

Also in lung and heart, elevated apoptotic cell numbers could be observed 9 days following ileitis induction in either cohort (*p* < 0.005 and *p* < 0.05 vs. naive, respectively; Figures 7A,D, Supplemental Figures S6A,D) that were accompanied by multi-fold increased T lymphocyte counts in both organs (*p* < 0.001; Figures 7B,E, Supplemental Figures S6B,E). Remarkably, increases in T cell numbers in lung and heart were more pronounced in Psae as compared to mock challenged mice (*p* < 0.01 and *p* < 0.05, respectively; Figures 7B,E, Supplemental Figures S6B,E). Only in the lung, but not in the heart, higher Treg numbers could be assessed at day 9 p.i. (*p* < 0.001; Figures 7C,F, Supplemental Figures S6C,F) with similar counts in either cohort. Hence, additional peroral Psae challenge resulted in increased apoptotic and T cell responses in extra-intestinal compartments.

### Extra-Intestinal Inflammatory Mediator Responses Upon MDR *P. aeruginosa* Challenge of Mice With a Human Gut Microbiota Suffering From Subacute Ileitis

**Figure 8:**
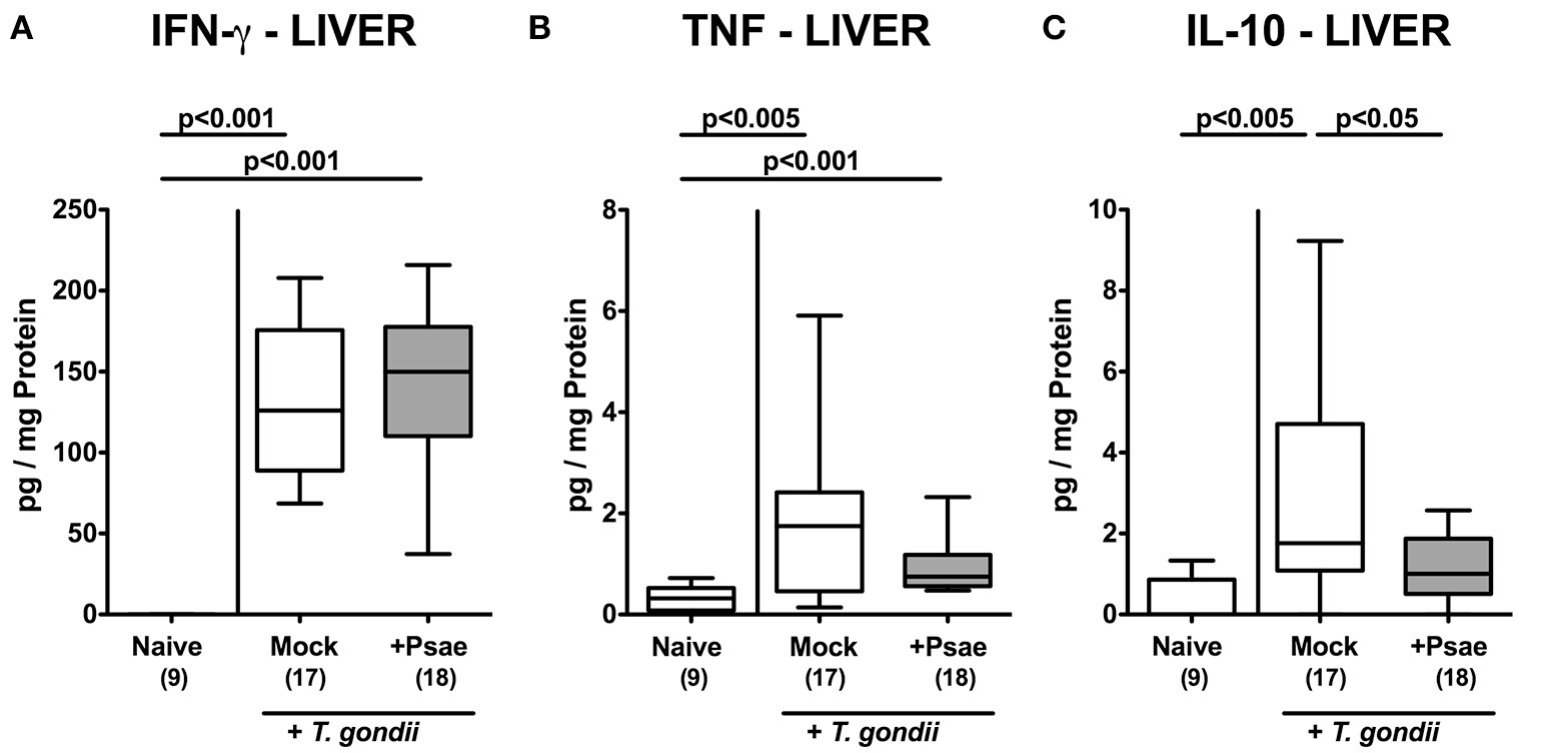
Pro- and anti-inflammatory mediator responses in the liver following peroral MDR *P. aeruginosa* challenge of mice with a human gut microbiota suffering from subacute ileitis. At day 5 following subacute ileitis induction, mice with a human gut microbiota were either perorally challenged with MDR *P. aeruginosa* (+Psae, dissolved in PBS) or with PBS alone (Mock). Uninfected mice with a human gut microbiota but without ileitis served as control animals (Naive). 96 h following Psae challenge, **(A)** IFN-γ, **(B)** TNF, and **(C)** IL-10 concentrations were determined in hepatic *ex vivo* biopsies. Box plots represent the 75th and 25th percentiles of the median (black bar inside the boxes). The total range and significance levels determined by one-way ANOVA test followed by Tukey post-correction test for multiple comparisons are shown. The total number of analyzed animals are given in parentheses. Data were pooled from four independent experiments.

We further assessed whether the observed increased apoptotic and immune cell responses in extra-intestinal compartments upon Psae challenge of hma mice with subacute ileitis were accompanied by pronounced inflammatory mediator secretion. Nine days following ileitis induction increased concentrations of pro-inflammatory cytokines such as IFN-γ and TNF could be observed in the liver of hma mice (*p* < 0.005–0.001; Figures 8A,B), but with no differences between mice of either cohort. Interestingly, hepatic concentrations of the anti-inflammatory cytokine IL-10 were elevated in mock treated, but not Psae challenged mice at day 9 p.i. (*p* < 0.005 vs. naive, *p* < 0.05 vs. Psae; Figure 8C).

In the kidney, pro-inflammatory cytokines including IFN-γ, TNF, MCP-1, and IL-6 were increased to similar extent in Psae and mock challenged mice at day 9 p.i. (*p* < 0.005–0.001 vs. naive; Supplemental Figure S7), which also held true for pulmonal IFN-γ, TNF, MCP-1, and IL-10 concentrations (*p* < 0.005–0.001 vs. naive; Supplemental Figure S8).

Hence, Psae challenge during subacute ileitis of hma mice did not result in further increased pro-inflammatory cytokine secretion in extra-intestinal organs, but led to a dampened anti-inflammatory IL-10 response in the liver.

### Systemic Pro-Inflammatory Mediator Responses Upon MDR *P. aeruginosa* Challenge of Mice With a Human Gut Microbiota Suffering From Subacute Ileitis

**Figure 9:**
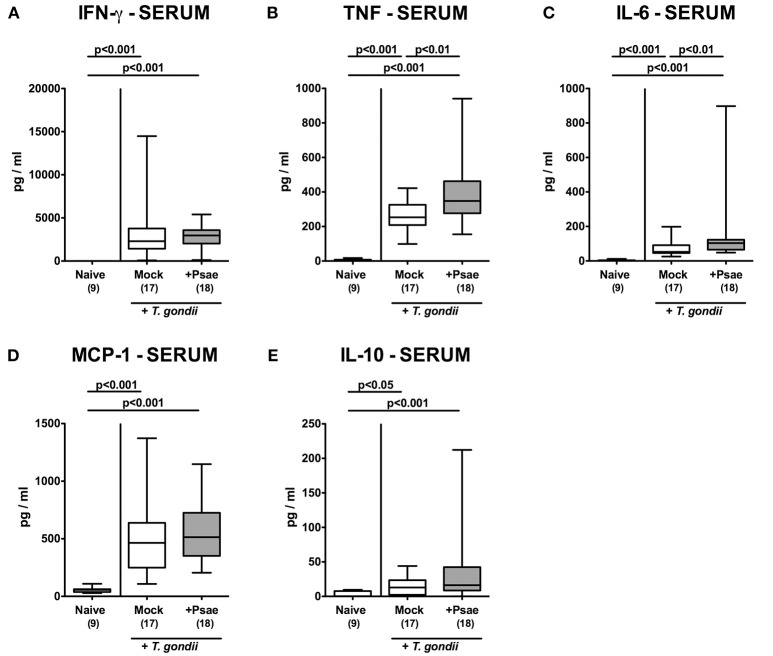
Systemic pro- and anti-inflammatory mediator responses following peroral MDR *P. aeruginosa* challenge of mice with a human gut microbiota suffering from subacute ileitis. At day 5 following subacute ileitis induction, mice with a human gut microbiota were either perorally challenged with MDR *P. aeruginosa* (+Psae, dissolved in PBS) or with PBS alone (Mock). Uninfected mice with a human gut microbiota but without ileitis served as control animals (Naive). 96 h following Psae challenge, **(A)** IFN-γ, **(B)** TNF, **(C)** IL-6, **(D)** MCP-1, and **(E)** IL-10 concentrations were determined in serum samples. Box plots represent the 75th and 25th percentiles of the median (black bar inside the boxes). The total range and significance levels determined by one-way ANOVA test followed by Tukey post-correction test for multiple comparisons are shown. The total number of analyzed animals are given in parentheses. Data were pooled from four independent experiments.

In the following we measured systemic cytokine secretion upon Psae challenge of hma mice during subacute ileitis. At day 9 p.i., increased pro-inflammatory mediators such as IFN-γ, TNF, and NO could be assessed in the spleen derived from mice of either cohort (*p* < 0.001; Supplemental Figure S9). In corresponding serum samples, increased IFN-γ, TNF, IL-6, MCP-1, and IL-10 concentrations were determined at day 9 p.i. (*p* < 0.05–0.001; Figure 9). Strikingly, even higher TNF and IL-6 levels could be measured in serum samples taken from Psae as compared to mock challenged hma mice with ileitis (*p* < 0.01; Figures 9 B,C). Of note, no viable bacteria that might have translocated from the intestinal tract to extra-intestinal and systemic tissue sites during subacute ileitis could be isolated from liver, kidney, lung, heart, spleen, or cardiac blood (not shown). Hence, Psae challenge of hma mice with induced subacute ileitis resulted in an augmented systemic pro-inflammatory cytokine response.

## Discussion

In recent years the global emergence of MDR Gram-negative species including Psae in humans as well as in livestock animals has come more and more into the focus of awareness (11, 30). In fact, the increasing prevalence of severe infections caused by MDR Gram-negative bacteria which allow for only very limited options to combat disease contributing to high fatality rates, has not only gained access to the conscience of medical professionals, but also to (health care) politicians as well as to the general public. In consequence and of utmost importance, the WHO rated MDR Gram-negative species such as Psae as serious global threat to human health recently (4).

In healthcare settings, the risk of a patient for MDR Psae acquisition increases with the length of the hospital stay, most likely due to spread of the opportunistic pathogen from external sources such as contaminated surfaces and medical devices and/or via smear infection from body fluids or fecal samples derived from colonized/infected patients (5, 6, 31). Particularly information regarding prevalence of human intestinal Psae carriage are scarce, however, and range from 0 to 24% in healthy individuals (31–40), hence speaking against Psae as part of the commensal gut microbiota. One of the key factors facilitating stable intestinal (opportunistic) pathogenic colonization including Psae in mice and men, however, is antibiotic pre-treatment compromising the integrity of the complex gut microbiota, which subsequently provides open niches for the acquired bacteria and hence, potential internal sources for MDR Psae-associated infections lateron (8, 9, 41, 42). In fact, fecal Psae carriage before ICU admission has been shown to be associated with an ~15-times increased risk for subsequent Psae-induced nosocomial infection (7), which might even more likely mount in a fatal outcome, given that particularly antibiotics-treated ICU patients with concomitant severe comorbidities including immunosuppression are prone to MDR Psae infection arising from the indigenous gut microbiota (3, 11, 43). It is therefore crucial to identify distinct risk factors including immunopathological conditions that might facilitate MDR Psae acquisition by the vertebrate host. Recent studies report higher abundances of Psae in the GIT of patients with underlying intestinal inflammatory diseases such as irritable bowel diseases or ulcerative colitis (40, 44). Interestingly, in our recent study MDR Psae could not only stably colonize the gut, but also induce overt inflammatory immune responses in intestinal, extra-intestinal and even systemic compartments upon peroral challenge of healthy WT mice that were lacking a gut microbiota due to preceding broad-spectrum antibiotic treatment (13). This was also the case, when (with respect to the gut microbiota) “humanized” and clinically uncompromised WT mice were subjected to the identical clinical MDR Psae strain (14).

In the present study, we had challenged hma mice with the opportunistic pathogen after subacute ileitis induction following peroral low-dose *T. gondii* infection—a non-lethal small intestinal inflammation model that has been established by us recently (19). Four days upon peroral application of a rather “supra-physiological” dose of 10^9^ CFU, Psae could be isolated from the intestinal tract in approximately one third of hma mice with subacute ileitis and if so, in relatively low counts of < 10^5^ CFU per g luminal sample. In our previous study, intestinal MDR Psae could be cultured in up to 78% of healthy hma mice (i.e., without underlying intestinal inflammatory conditions) 4 weeks following peroral challenge with comparable loads (14). In line with results obtained from our mouse studies, fecal Psae counts were up to 5 log orders of magnitude lower in healthy human volunteers as compared to the bacterial counts within the oral suspension 6 days before (12).

Remarkably, irrespective of the (opportunistic) pathogenic bacterial carrier status, Psae-challenged mice appeared to display more pronounced clinical/macroscopic and microscopic features of subacute ileitis. Whereas, 72.2% of mice from the Psae cohort displayed macroscopic and/or microscopic abundance of blood in their feces 4 day after Psae challenge, this was only the case in 58.8% of mock counterparts. In support, the histopathological changes in the ileal mucosa tended to be more severe in the Psae cohort as compared to the mock controls, given that individual mice exhibited even virtually fatal ileal mucosal changes such as transmural necrosis 4 days post Psae challenge, which could not be observed in the mock counterparts. The relatively high standard deviation in histopathological scores within the MDR Psae cohort points toward (yet unknown) distinct host factors that might determine the individual outcome of subacute ileitis upon Psae infection.

Furthermore, Psae induced a marked influx of T lymphocytes into the mucosa and lamina propria of both, the ileum and the colon that was accompanied by pronounced apoptotic responses in the ileal epithelia. Remarkably, the marked Psae-induced T cell responses were not restricted to the intestinal tract, but could also be observed in extra-intestinal compartments such as liver, kidney, lungs, and even the heart. In the liver, significant Psae-induced apoptotic changes could be further assessed and were accompanied by less distinct secretion of the anti-inflammatory cytokine IL-10 as compared to mock control mice. Strikingly, Psae challenged hma mice with subacute ileitis displayed even more pronounced systemic pro-inflammatory cytokine responses versus mock counterparts as indicated by higher TNF and IL-6 serum concentrations in the former as compared to the latter. In this context we were, however, not able to detect any viable bacteria originating from the gut microbiota including Psae that had translocated to extra-intestinal including systemic compartments. Nevertheless, it cannot be excluded that soluble bacterial factors including lipopolysaccharide derived from Gram-negative intestinal commensals or from MDR Psae might have found access to the circulation evoking systemic pro-inflammatory immune responses.

In support of our actual results, we demonstrated previously that peroral MDR Psae application to conventionally colonized IL-10^−/−^ mice suffering from chronic colitis aggravated the outcome of the underlying intestinal immunopathological disease (16), again not only on the local (i.e., intestinal) level, but also beyond (i.e., in extra-intestinal and even systemic compartments).

Another recent study applying a lethal acute ileitis model induced by peroral high-dose (i.e., >50 cysts) *T. gondii* infection of hma mice revealed that the underlying acute gut inflammation facilitated Psae colonization (15). The severity of the gut inflammation model was, however, far too acute to decipher additional Psae induced inflammatory sequelae further accelerating the underlying (already hyper-acute) immunopathology (15).

Nevertheless, given the multitude of so far known Psae virulence factor it is not surprising that intestinal Psae carriage in health and disease mounts in immune responses accelerating the underlying disease. The *Pseudomonas* lipid A moiety, for instance, is part of the Gram-negative bacterial cell wall constituent lipopeptide and able to activate NFκB in a Toll-like receptor (TLR)−4 dependent fashion (45), which subsequently leads to recruitment of innate immune cells to the infection site, further perpetuating host immune responses in order to combat the (opportunistic) pathogenic challenge (1). In support, our previous studies revealed that, in fact, TLR-4 was involved in differentially mediating MDR Psae associated intestinal and extra-intestinal immune responses in IL-10^−/−^ mice (46, 47).

The observed Psae induced immunopathological sequelae in hma mice with subacute ileitis -irrespective of the intestinal carrier status—are supported by our results derived from *Campylobacter jejuni* infection experiments indicating that a pathogen does not necessarily needs to be a permanent member of the intestinal ecosystem to induce pro-inflammatory responses (26, 48–50). More importantly, it is rather the initial hit set by the (opportunistic) pathogen that tips the balance toward immunopathology within the “ménage à trois” of (opportunistic) pathogens, commensal gut microbiota and host immunity (47, 48).

We further assessed whether Psae application resulted in changes of the gut microbiota composition applying culture-independent methods, but could neither find any significant and biologically relevant Psae-induced changes in the luminal microbiota of the inflamed ileum, nor in the co-affected colon. One might, in fact, argue that the observation period of 4 days was far too short for overt changes in the intestinal ecosystem. Nevertheless, our actual results are supported by our previous study where even an intestinal carriage of MDR Psae by conventional IL-10^−/−^ mice with chronic colitis for 6 weeks was not associated with significant changes in the gut microbiota composition as assessed by both, culture and molecular analyses (16).

In conclusion, our actual and previous studies revealed that peroral exposure to MDR Gram-negative opportunistic pathogenic species including Psae might worsen the outcome of underlying inflammatory conditions within the gut. In ongoing studies we are currently exploring antibiotics-independent factors preventing the susceptible host at risk including individuals suffering from inflammatory intestinal comorbidities from Psae-carriage and induced inflammation.

## Author Contributions

MH: designed and performed experiments, analyzed data, wrote paper; UE and AG: performed experiments, analyzed data, co-edited paper; AK: analyzed data, co-edited paper; SB: provided advice in design and performance of experiments, co-edited paper.

### Conflict of Interest Statement

The authors declare that the research was conducted in the absence of any commercial or financial relationships that could be construed as a potential conflict of interest.

## Supplementary Material

Supplemental Figure S1 Experimental setup. The gut microbiota was depleted in conventional mice by broad-spectrum antibiotic treatment for 8 weeks. Three days before human fecal microbiota transplantation (FMT) the antibiotic cocktail was replaced by sterile tap water to assure antibiotic washout. Microbiota depleted animals were then subjected to human FMT on three consecutive days. In order to guarantee proper establishment of the complex human gut microbiota within the murine host, mice were kept for 10 days before subacute ileitis induction. At day 0, human microbiota associated (hma) mice were perorally subjected to low dose (i.e., one cyst) infection with *T. gondii* infection. 5 days later (d+5), hma mice with induced subacute ileitis were perorally challenged with a multi-drug resistant (MDR) *P. aeruginosa* (Psae) strain and followed up for another 4 days (d+9; necropsy).

Supplemental Figure S2 Microbiota composition of human donor feces. Before human fecal microbiota transplantation of secondary abiotic mice on three consecutive days, main intestinal bacterial groups were quantitatively assessed in human fecal donor suspensions. 16S rRNA of the main intestinal bacterial commensals including enterobacteria (EB), enterococci (EC), lactobacilli (LB), bifidobacteria (Bif), *Bacteroides / Prevotella* species (B/P), *Clostridium coccoides* group (Clocc), *Clostridium leptum* group (Clept), and the total eubacterial load (TL) were analyzed by quantitative RT-PCR and expressed as gene numbers per ng DNA. Data shown are representative for four independent experiments. Total range as well as box plots representing the 75th and 25th percentiles of the median (black bar inside the boxes) are shown.

Supplemental Figure S3 Representative photomicrographs depicting apoptotic and proliferating epithelial cell as well as immune cell responses in the ileum and colon following peroral MDR *P. aeruginosa* challenge of mice with a human gut microbiota suffering from subacute ileitis. At day 5 following subacute ileitis induction, mice with a human gut microbiota were either perorally challenged with MDR *P. aeruginosa* (ILE, Psae; dissolved in PBS) or with PBS alone (ILE, Mock). Uninfected mice with a human gut microbiota but without ileitis served as control animals (Naive). Representative photomicrographs out of four independent experiments illustrate the average numbers of apoptotic (Casp3+; **A,E**) and proliferating (Ki67+; **B,F**) epithelial cells as well as of T lymphocytes (CD3+; **C,G**) and regulatory T cells (FOXP3+; **D,H**) in at least six high power fields (HPF) that had been quantitatively assessed in ileal **(A–D)** and colonic **(E–H)** paraffin sections 96 h following Psae challenge applying *in situ* immunhistochemistry (100x magnification; scale bar: 100 μm).

Supplemental Figure S4 Intestinal pro-inflammatory mediator responses following peroral MDR *P. aeruginosa* challenge of mice with a human gut microbiota suffering from subacute ileitis. At day 5 following subacute ileitis induction, mice with a human gut microbiota were either perorally challenged with MDR *P. aeruginosa* (+Psae, dissolved in PBS) or with PBS alone (Mock). Uninfected mice with a human gut microbiota but without ileitis served as control animals (Naive). 96 h following Psae challenge, IFN-γ concentrations were measured in *ex vivo* biopsies derived from the **(A)** ileum, **(B)** colon, and **(C)** mesenteric lymph nodes (MLN). In addition, **(D)** TNF and **(E)** nitric oxide levels were determined in MLN *ex vivo* biopsies. Box plots represent the 75th and 25th percentiles of the median (black bar inside the boxes). The total range and significance levels determined by one-way ANOVA test followed by Tukey post-correction test for multiple comparisons are shown. The total numbers of analyzed animals are given in parentheses. Data were pooled from four independent experiments.

Supplemental Figure S5 Representative photomicrographs depicting apoptotic epithelial cell as well as immune cell responses in the liver and kidney following peroral MDR *P. aeruginosa* challenge of mice with a human gut microbiota suffering from subacute ileitis. At day 5 following subacute ileitis induction, mice with a human gut microbiota were either perorally challenged with MDR *P. aeruginosa* (ILE, Psae; dissolved in PBS) or with PBS alone (ILE, Mock). Uninfected mice with a human gut microbiota but without ileitis served as control animals (Naive). Representative photomicrographs out of four independent experiments illustrate the average numbers of apoptotic (Casp3+; **A,D**) epithelial cells as well as of T lymphocytes (CD3+; **B,E**) and regulatory T cells (FOXP3+; **C,F**) in at least six high power fields (HPF) that had been were quantitatively assessed in hepatic **(A–C)** and renal **(D–F)** paraffin sections 96 h following Psae challenge applying *in situ* immunhistochemistry (100x magnification; scale bar: 100 μm).

Supplemental Figure S6 Representative photomicrographs depicting apoptotic epithelial cell as well as immune cell responses in the lung and heart following peroral MDR *P. aeruginosa* challenge of mice with a human gut microbiota suffering from subacute ileitis. At day 5 following subacute ileitis induction, mice with a human gut microbiota were either perorally challenged with MDR *P. aeruginosa* (ILE, Psae; dissolved in PBS) or with PBS alone (ILE, Mock). Uninfected mice with a human gut microbiota but without ileitis served as control animals (Naive). Representative photomicrographs out of four independent experiments illustrate the average numbers of apoptotic (Casp3+; **A,D**) epithelial cells as well as of T lymphocytes (CD3+; **B,E**) and regulatory T cells (FOXP3+; **C,F**) in at least six high power fields (HPF) that had been were quantitatively assessed in pulmonal **(A–C)** and cardiac **(D–F)** paraffin sections 96 h following Psae challenge applying *in situ* immunhistochemistry (100x magnification; scale bar: 100 μm).

Supplemental Figure S7 Cytokine responses in the kidneys following peroral MDR *P. aeruginosa* challenge of mice with a human gut microbiota suffering from subacute ileitis. At day 5 following subacute ileitis induction, mice with a human gut microbiota were either perorally challenged with MDR *P. aeruginosa* (+Psae, dissolved in PBS) or with PBS alone (Mock). Uninfected mice with a human gut microbiota but without ileitis served as control animals (Naive). 96 h following Psae challenge, **(A)** IFN-γ, **(B)** TNF, **(C)** MCP-1, and **(D)** IL-6 concentrations were determined in renal *ex vivo* biopsies. Box plots represent the 75th and 25th percentiles of the median (black bar inside the boxes). The total range and significance levels determined by one-way ANOVA test followed by Tukey post-correction test for multiple comparisons are shown. The total numbers of analyzed animals are given in parentheses. Data were pooled from four independent experiments.

Supplemental Figure S8 Cytokine responses in the lungs following peroral MDR *P. aeruginosa* challenge of mice with a human gut microbiota suffering from subacute ileitis. At day 5 following subacute ileitis induction, mice with a human gut microbiota were either perorally challenged with MDR *P. aeruginosa* (+Psae, dissolved in PBS) or with PBS alone (Mock). Uninfected mice with a human gut microbiota but without ileitis served as control animals (Naive). 96 h following Psae challenge, **(A)** IFN-γ, **(B)** TNF, **(C)** MCP-1, and **(D)** IL-10 concentrations were determined in pulmonal *ex vivo* biopsies. Box plots represent the 75th and 25th percentiles of the median (black bar inside the boxes). The total range and significance levels determined by one-way ANOVA test followed by Tukey post-correction test for multiple comparisons are shown. The total numbers of analyzed animals are given in parentheses. Data were pooled from four independent experiments.

Supplemental Figure S9 Pro-inflammatory mediator responses in the spleen following peroral MDR *P. aeruginosa* challenge of mice with a human gut microbiota suffering from subacute ileitis. At day 5 following subacute ileitis induction, mice with a human gut microbiota were either perorally challenged with MDR *P. aeruginosa* (+Psae, dissolved in PBS) or with PBS alone (Mock). Uninfected mice with a human gut microbiota but without ileitis served as control animals (Naive). 96 h following Psae challenge, **(A)** IFN-γ, **(B)** TNF, and **(C)** nitric oxide concentrations were determined in splenic *ex vivo* biopsies. Box plots represent the 75th and 25th percentiles of the median (black bar inside the boxes). The total range and significance levels determined by one-way ANOVA test followed by Tukey post-correction test for multiple comparisons are shown. The total numbers of analyzed animals are given in parentheses. Data were pooled from four independent experiments.

## Abbreviations

CFU: colony forming units
FMT: fecal microbiota transplantation
GIT: gastrointestinal tract
H&E: hematoxylin and eosin
hma: human microbiota associated
HPF: high power field
ICU: intensive care unit
MDR: multidrug-resistant
MLN: mesenteric lymph nodes
NO: nitric oxide
PBS: phosphate buffered saline
Psae: *Pseudomonas aeruginosa*
SPF: specific pathogen free
TLR: Toll-like receptor
Treg: regulatory T cells
WHO: World Health Organization
WT: wildtype
