# Supplementary figures and images for: Multidrug-Resistant Pseudomonas aeruginosa Accelerate Intestinal, Extra-Intestinal, and Systemic Inflammatory Responses in Human Microbiota-Associated Mice With Subacute Ileitis
Source: Front Immunol. 2019 Jan 29;10:49. doi: 10.3389/fimmu.2019.00049 (PMC6361842; doi:10.3389/fimmu.2019.00049)

# Experimental Setup

Antibiotic Cocktail      human FMT      *T. gondii*      MDR *Psae*      Necropsy

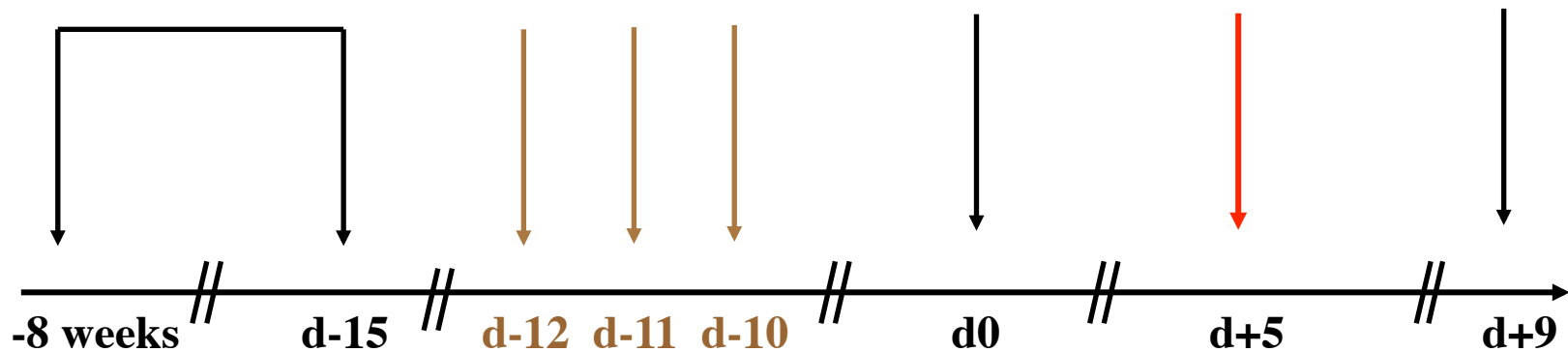

Supplement: Supplemental Figure S1 — Experimental setup. The gut microbiota was depleted in conventional mice by broad-spectrum antibiotic treatment for 8 weeks. Three days before human fecal microbiota transplantation (FMT) the antibiotic cocktail was replaced by sterile tap water to assure antibiotic washout. Microbiota depleted animals were then subjected to human FMT on three consecutive days. In order to guarantee proper establishment of the complex human gut microbiota within the murine host, mice were kept for 10 days before subacute ileitis induction. At day 0, human microbiota associated (hma) mice were perorally subjected to low dose (i.e., one cyst) infection with T. gondii infection. 5 days later (d+5), hma mice with induced subacute ileitis were perorally challenged with a multi-drug resistant (MDR) P. aeruginosa (Psae) strain and followed up for another 4 days (d+9; necropsy). [file Image_1.pdf]

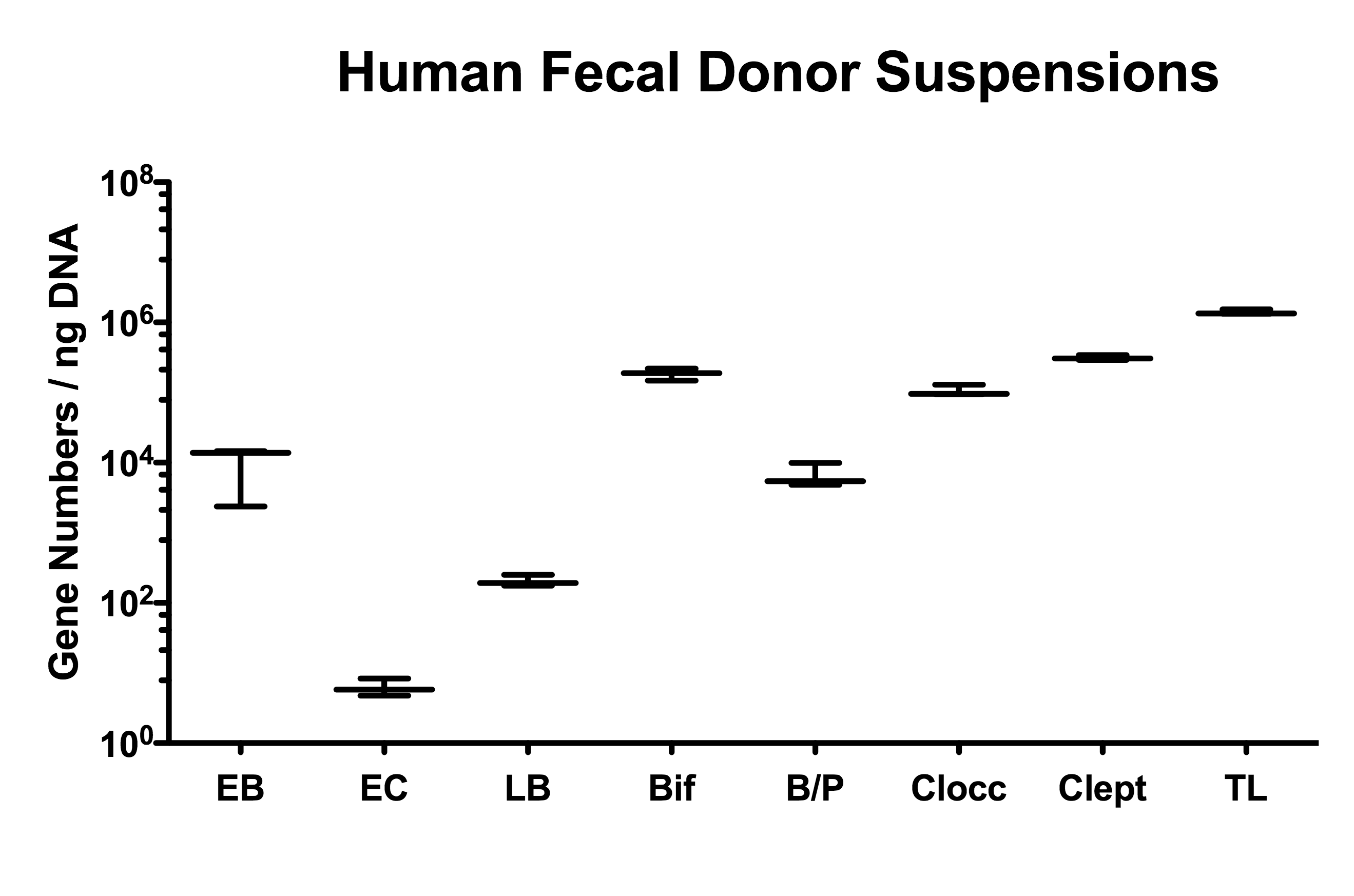

Supplement: Supplemental Figure S2 — Microbiota composition of human donor feces. Before human fecal microbiota transplantation of secondary abiotic mice on three consecutive days, main intestinal bacterial groups were quantitatively assessed in human fecal donor suspensions. 16S rRNA of the main intestinal bacterial commensals including enterobacteria (EB), enterococci (EC), lactobacilli (LB), bifidobacteria (Bif), Bacteroides / Prevotella species (B/P), Clostridium coccoides group (Clocc), Clostridium leptum group (Clept), and the total eubacterial load (TL) were analyzed by quantitative RT-PCR and expressed as gene numbers per ng DNA. Data shown are representative for four independent experiments. Total range as well as box plots representing the 75th and 25th percentiles of the median (black bar inside the boxes) are shown. [file Image_2.tiff]

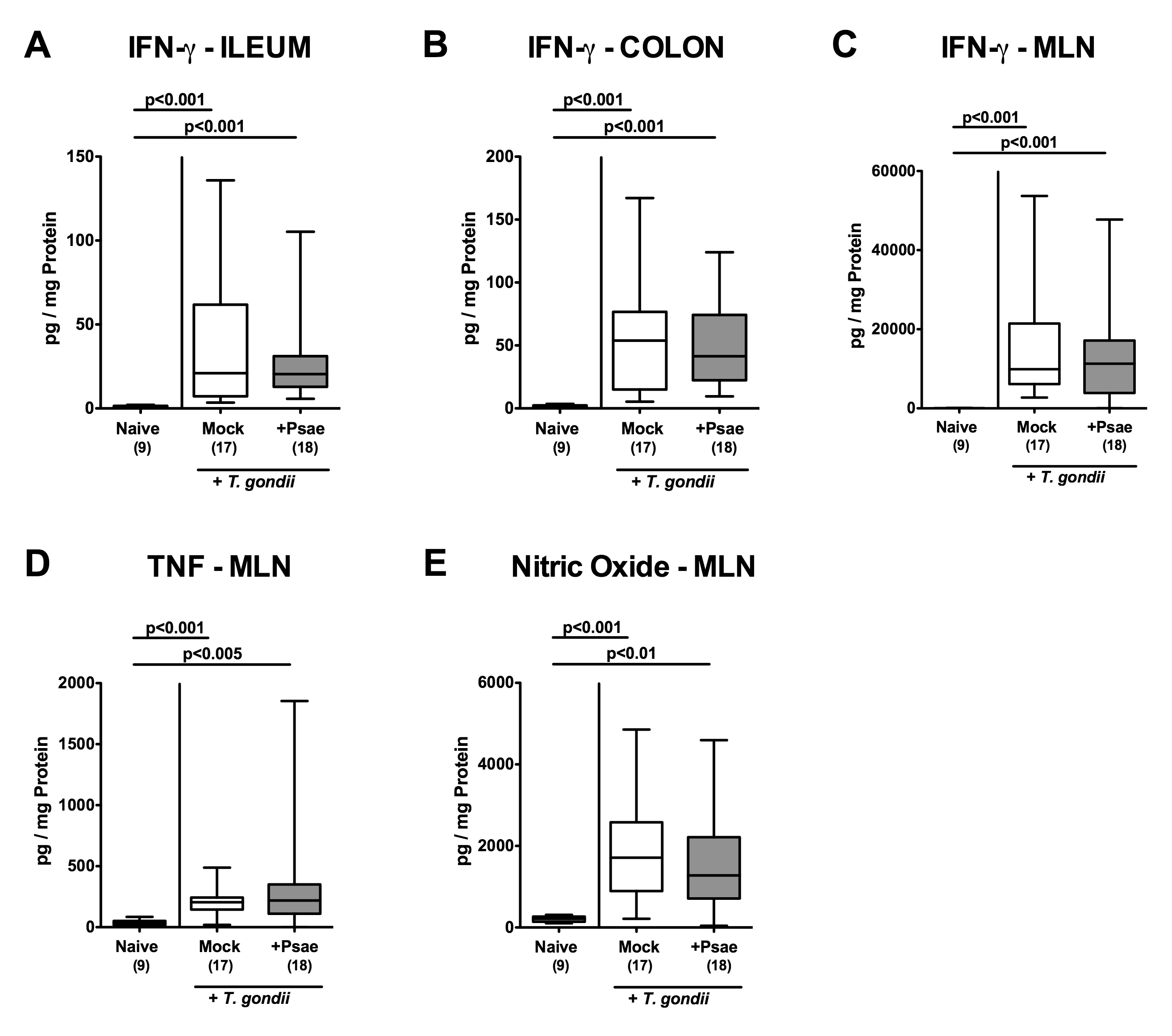

Supplement: Supplemental Figure S4 — Intestinal pro-inflammatory mediator responses following peroral MDR P. aeruginosa challenge of mice with a human gut microbiota suffering from subacute ileitis. At day 5 following subacute ileitis induction, mice with a human gut microbiota were either perorally challenged with MDR P. aeruginosa (+Psae, dissolved in PBS) or with PBS alone (Mock). Uninfected mice with a human gut microbiota but without ileitis served as control animals (Naive). 96 h following Psae challenge, IFN-γ concentrations were measured in ex vivo biopsies derived from the (A) ileum, (B) colon, and (C) mesenteric lymph nodes (MLN). In addition, (D) TNF and (E) nitric oxide levels were determined in MLN ex vivo biopsies. Box plots represent the 75th and 25th percentiles of the median (black bar inside the boxes). The total range and significance levels determined by one-way ANOVA test followed by Tukey post-correction test for multiple comparisons are shown. The total numbers of analyzed animals are given in parentheses. Data were pooled from four independent experiments. [file Image_4.tiff]

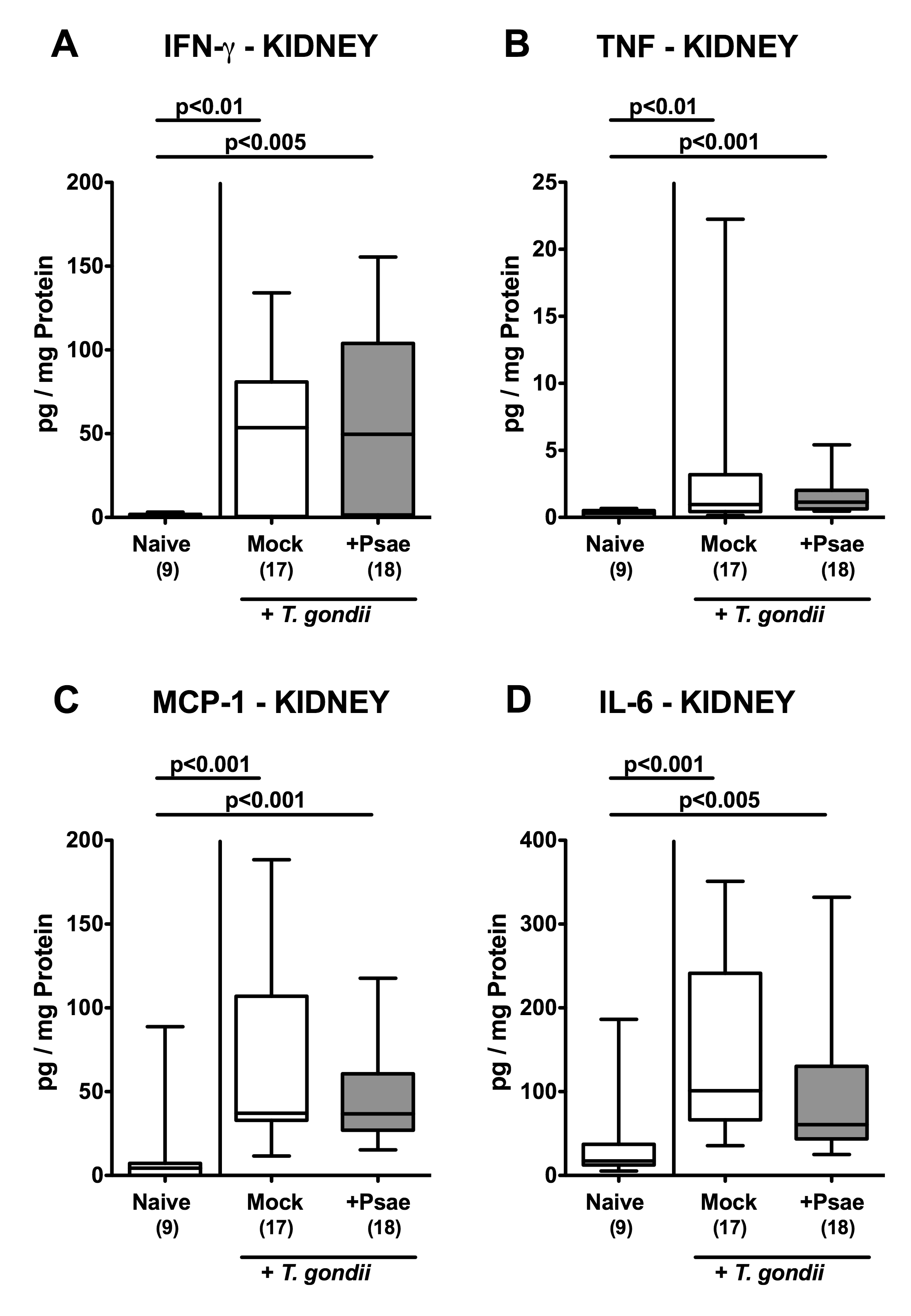

Supplement: Supplemental Figure S7 — Cytokine responses in the kidneys following peroral MDR P. aeruginosa challenge of mice with a human gut microbiota suffering from subacute ileitis. At day 5 following subacute ileitis induction, mice with a human gut microbiota were either perorally challenged with MDR P. aeruginosa (+Psae, dissolved in PBS) or with PBS alone (Mock). Uninfected mice with a human gut microbiota but without ileitis served as control animals (Naive). 96 h following Psae challenge, (A) IFN-γ, (B) TNF, (C) MCP-1, and (D) IL-6 concentrations were determined in renal ex vivo biopsies. Box plots represent the 75th and 25th percentiles of the median (black bar inside the boxes). The total range and significance levels determined by one-way ANOVA test followed by Tukey post-correction test for multiple comparisons are shown. The total numbers of analyzed animals are given in parentheses. Data were pooled from four independent experiments. [file Image_7.tiff]

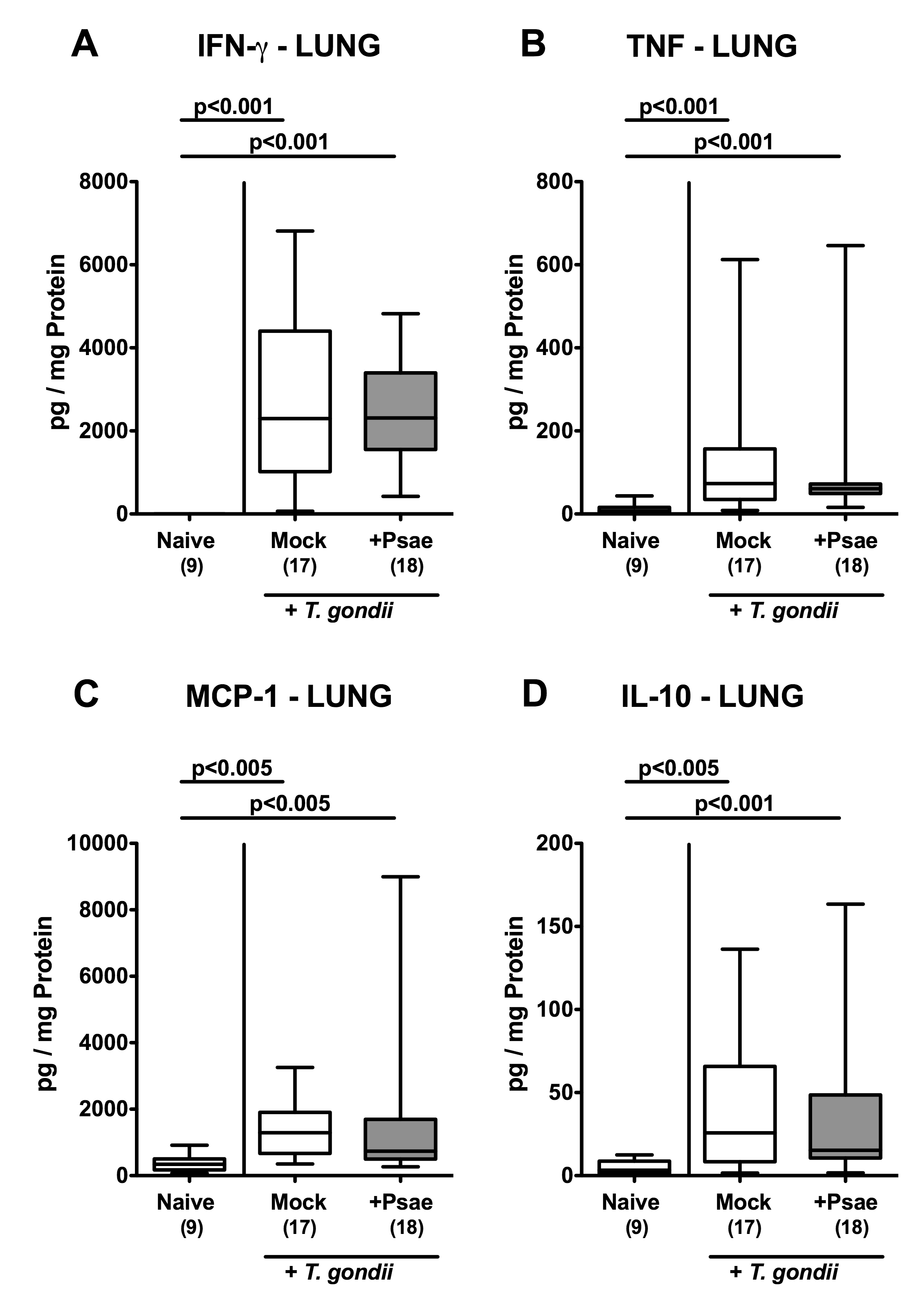

Supplement: Supplemental Figure S8 — Cytokine responses in the lungs following peroral MDR P. aeruginosa challenge of mice with a human gut microbiota suffering from subacute ileitis. At day 5 following subacute ileitis induction, mice with a human gut microbiota were either perorally challenged with MDR P. aeruginosa (+Psae, dissolved in PBS) or with PBS alone (Mock). Uninfected mice with a human gut microbiota but without ileitis served as control animals (Naive). 96 h following Psae challenge, (A) IFN-γ, (B) TNF, (C) MCP-1, and (D) IL-10 concentrations were determined in pulmonal ex vivo biopsies. Box plots represent the 75th and 25th percentiles of the median (black bar inside the boxes). The total range and significance levels determined by one-way ANOVA test followed by Tukey post-correction test for multiple comparisons are shown. The total numbers of analyzed animals are given in parentheses. Data were pooled from four independent experiments. [file Image_8.tiff]

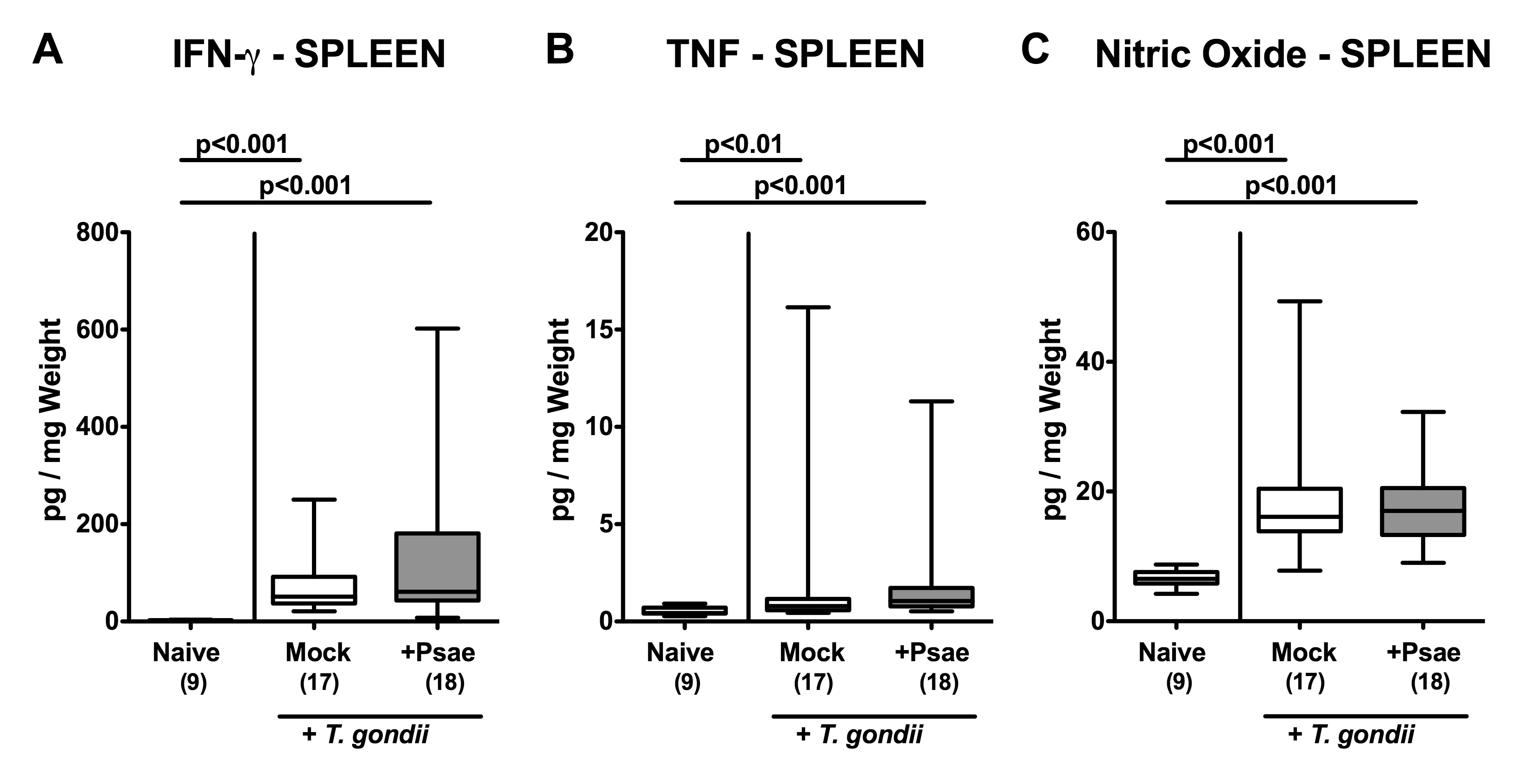

Supplement: Supplemental Figure S9 — Pro-inflammatory mediator responses in the spleen following peroral MDR P. aeruginosa challenge of mice with a human gut microbiota suffering from subacute ileitis. At day 5 following subacute ileitis induction, mice with a human gut microbiota were either perorally challenged with MDR P. aeruginosa (+Psae, dissolved in PBS) or with PBS alone (Mock). Uninfected mice with a human gut microbiota but without ileitis served as control animals (Naive). 96 h following Psae challenge, (A) IFN-γ, (B) TNF, and (C) nitric oxide concentrations were determined in splenic ex vivo biopsies. Box plots represent the 75th and 25th percentiles of the median (black bar inside the boxes). The total range and significance levels determined by one-way ANOVA test followed by Tukey post-correction test for multiple comparisons are shown. The total numbers of analyzed animals are given in parentheses. Data were pooled from four independent experiments. [file Image_9.tiff]
