# Supplementary material for: Multidrug-Resistant Pseudomonas aeruginosa Accelerate Intestinal, Extra-Intestinal, and Systemic Inflammatory Responses in Human Microbiota-Associated Mice With Subacute Ileitis
Source: Front Immunol. 2019 Jan 29;10:49. doi: 10.3389/fimmu.2019.00049 (PMC6361842; doi:10.3389/fimmu.2019.00049)

A

Apoptotic Cells (Casp3+) - LIVER

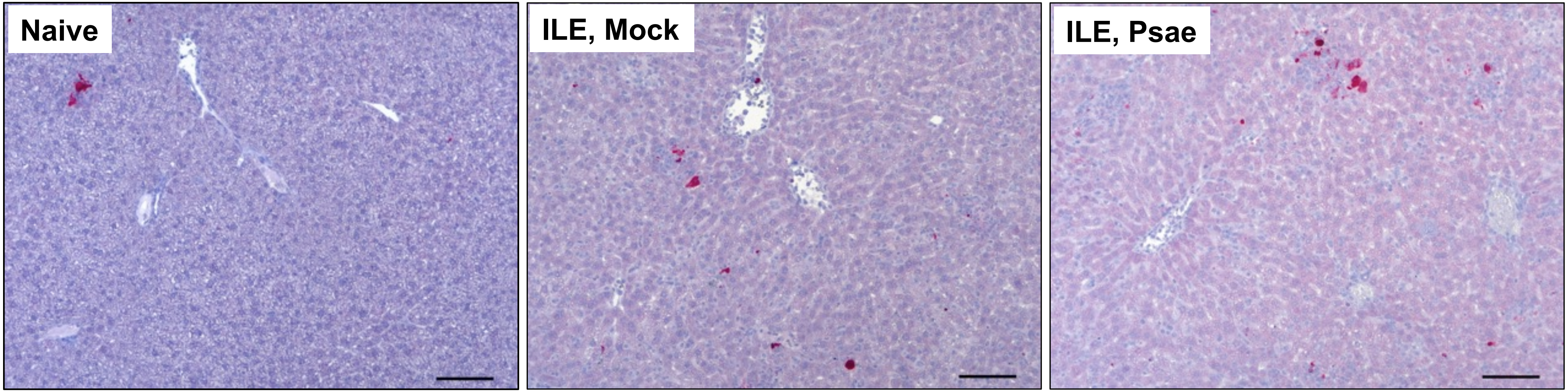

B

T Lymphocytes (CD3+) - LIVER

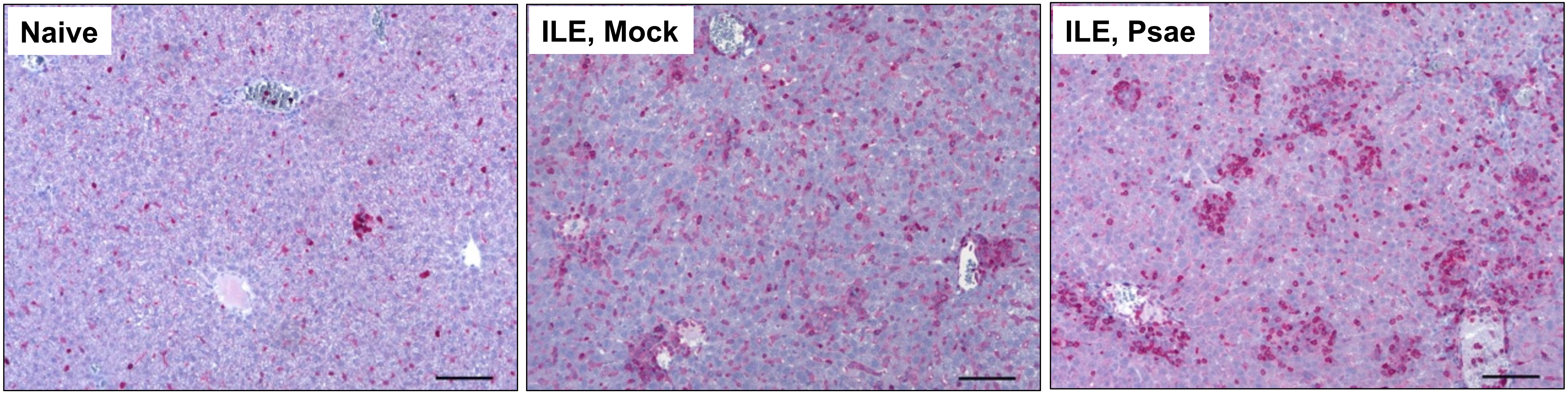

C

Regulatory T Cells (Treg; FOXP3+) - LIVER

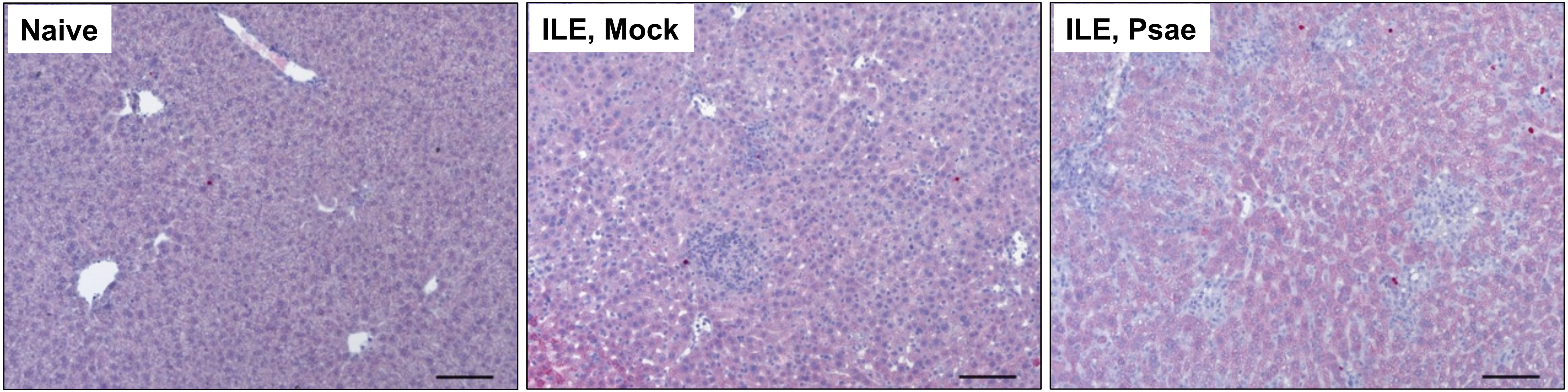

D

Apoptotic Cells (Casp3+) - KIDNEY

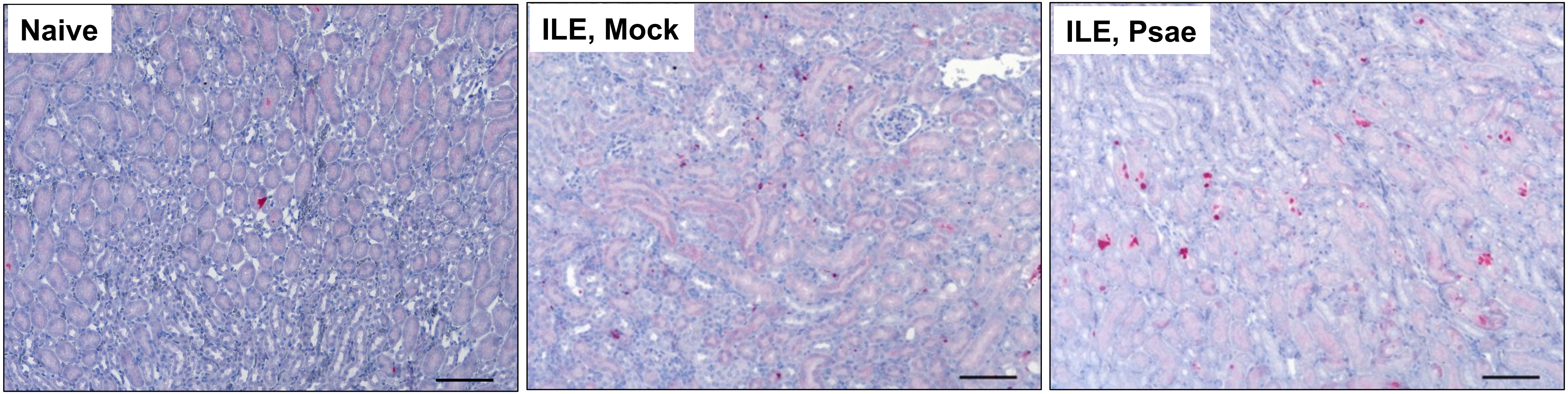

E

T Lymphocytes (CD3+) - KIDNEY

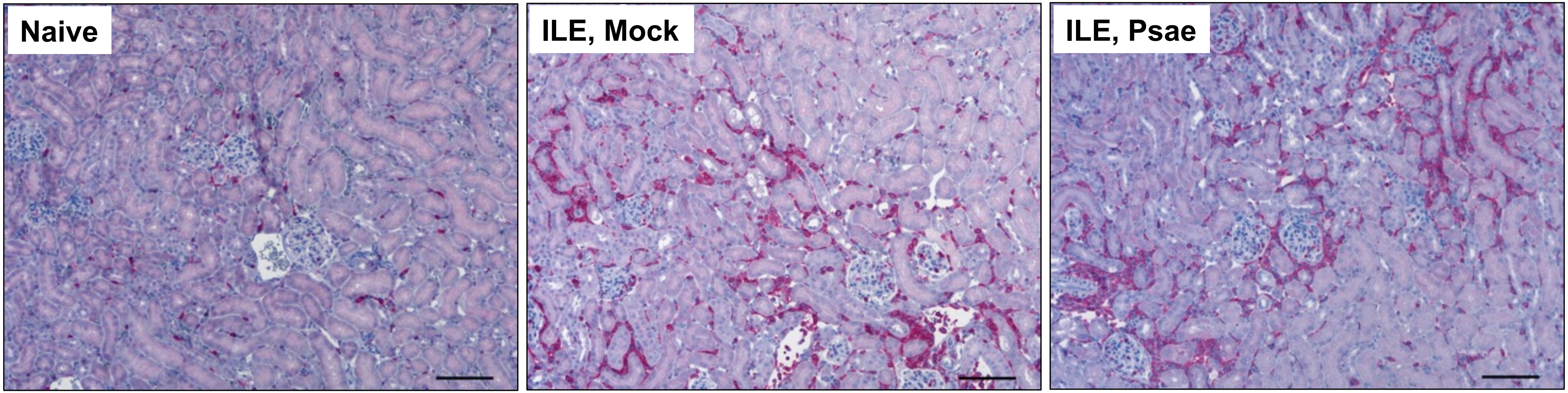

F

Regulatory T Cells (Treg; FOXP3+) - KIDNEY

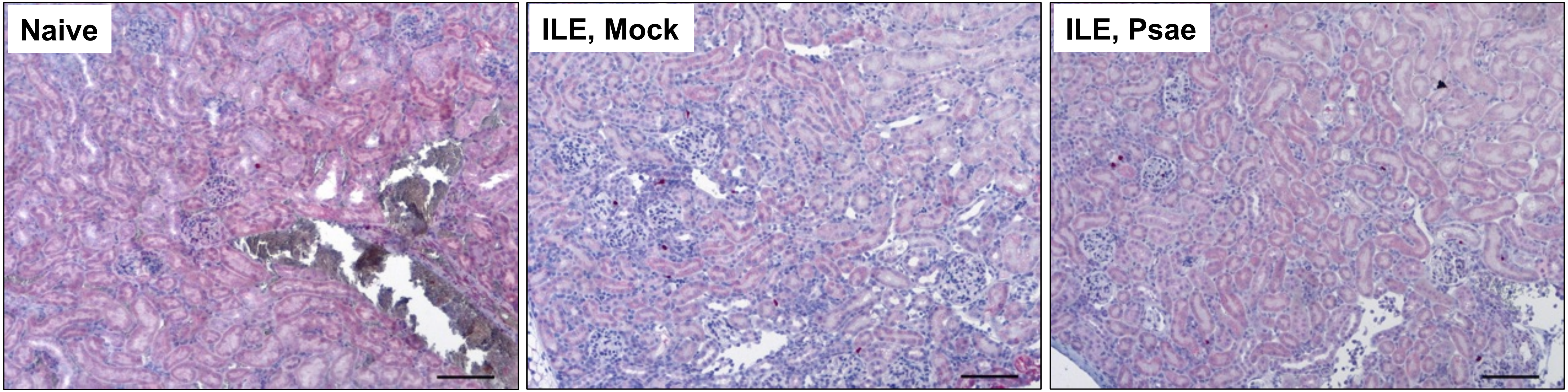

Apoptotic Cells (Casp3+) - LUNG

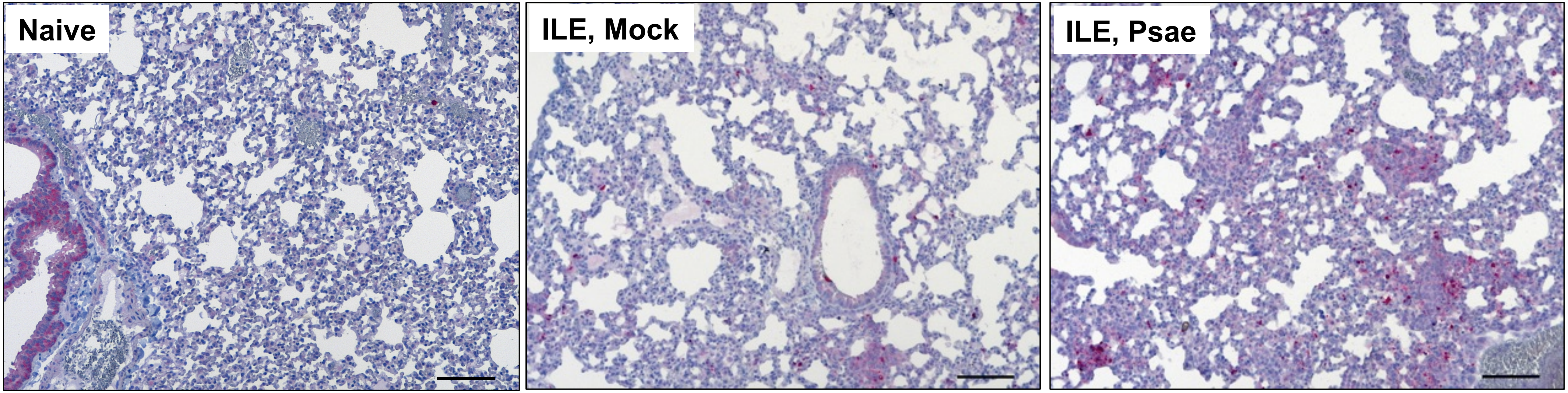

T Lymphocytes (CD3+) - LUNG

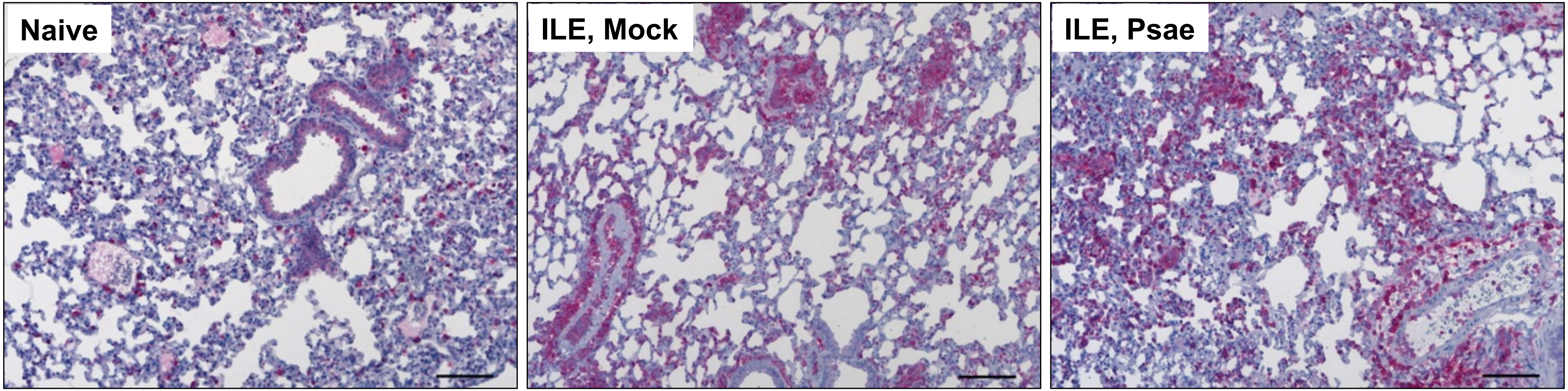

Regulatory T Cells (Treg; FOXP3+) - LUNG

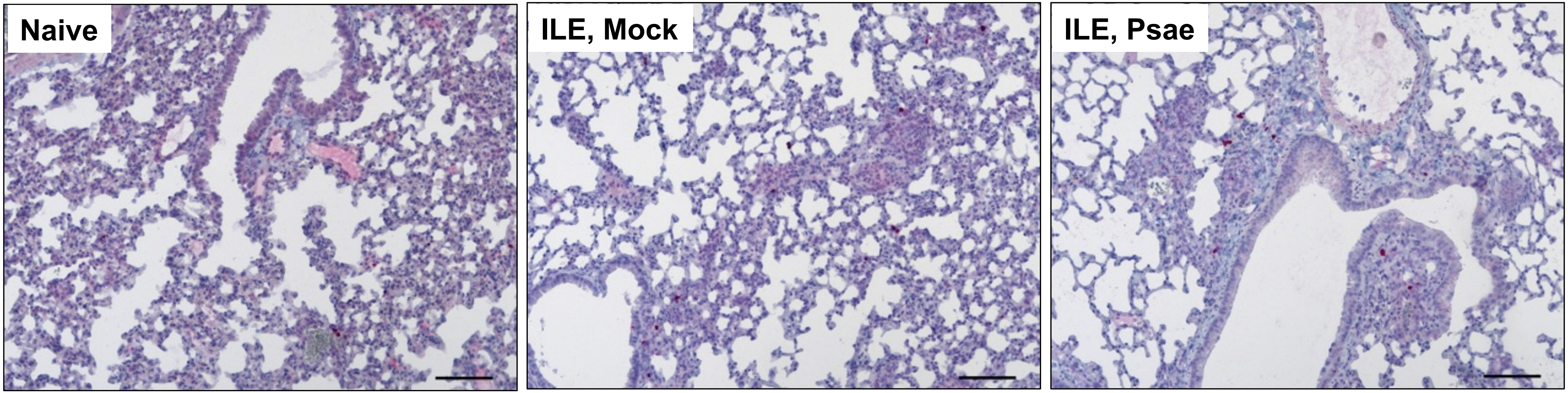

Apoptotic Cells (Casp3+) - HEART

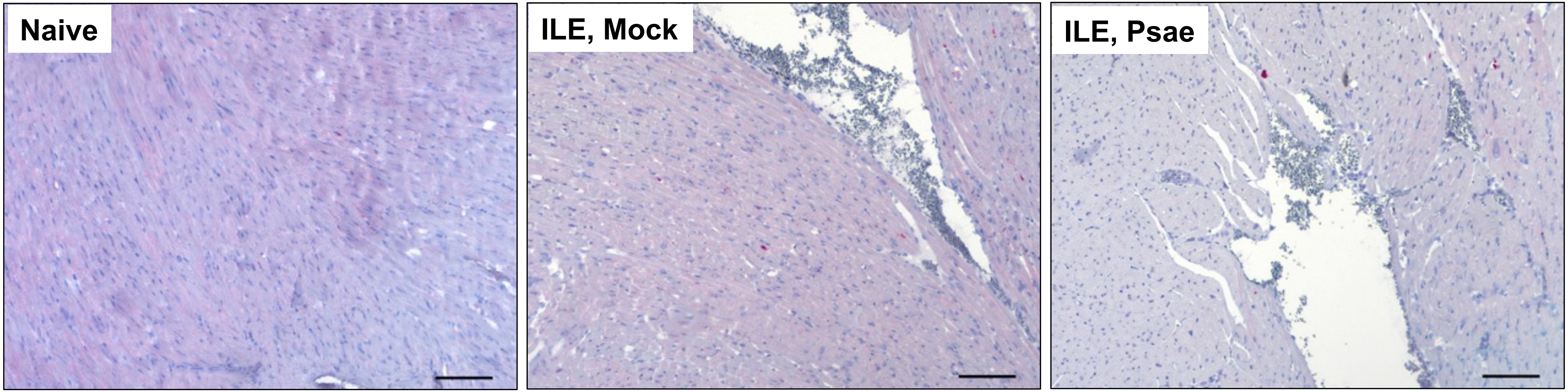

T Lymphocytes (CD3+) - HEART

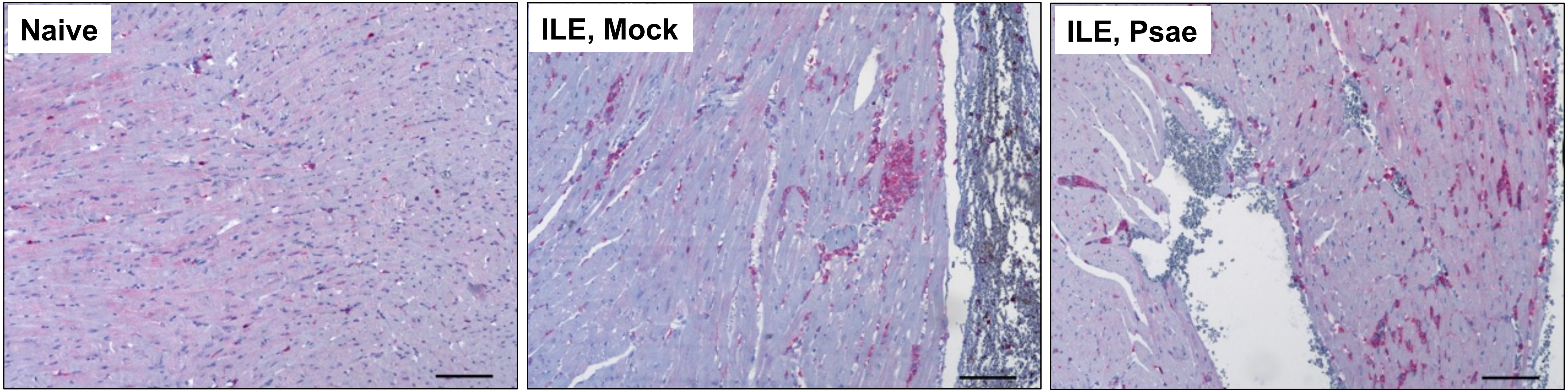

Regulatory T Cells (Treg; FOXP3+) - HEART

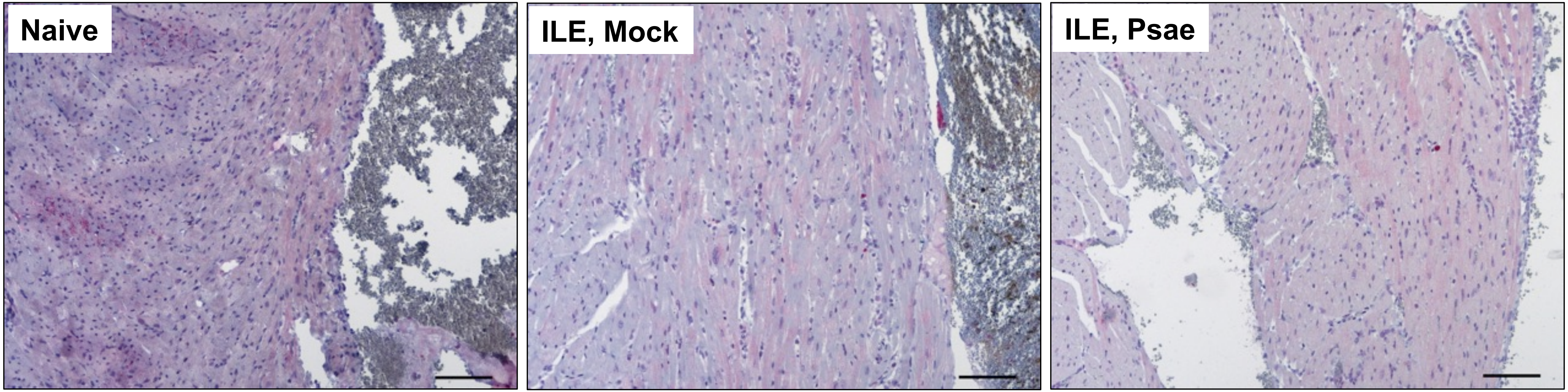

Supplement: Supplemental Figure S5 — Representative photomicrographs depicting apoptotic epithelial cell as well as immune cell responses in the liver and kidney following peroral MDR P. aeruginosa challenge of mice with a human gut microbiota suffering from subacute ileitis. At day 5 following subacute ileitis induction, mice with a human gut microbiota were either perorally challenged with MDR P. aeruginosa (ILE, Psae; dissolved in PBS) or with PBS alone (ILE, Mock). Uninfected mice with a human gut microbiota but without ileitis served as control animals (Naive). Representative photomicrographs out of four independent experiments illustrate the average numbers of apoptotic (Casp3+; A,D) epithelial cells as well as of T lymphocytes (CD3+; B,E) and regulatory T cells (FOXP3+; C,F) in at least six high power fields (HPF) that had been were quantitatively assessed in hepatic (A–C) and renal (D–F) paraffin sections 96 h following Psae challenge applying in situ immunhistochemistry (100x magnification; scale bar: 100 μm). [file Image_5.pdf]
